# The effects of nutritional supplementation on older sarcopenic individuals who engage in resistance training: a meta-analysis

**DOI:** 10.3389/fnut.2023.1109789

**Published:** 2023-04-25

**Authors:** Zixian Song, Tingting Pan, Xu Tong, Ying Yang, Ze Zhang

**Affiliations:** ^1^Department of Internal Medicine, First Clinical College, Liaoning University of Traditional Chinese Medicine, Shenyang, China; ^2^Department of Geriatrics, Affiliated Hospital of Liaoning University of Traditional Chinese Medicine, Shenyang, China

**Keywords:** sarcopenia, nutrition, resistance training, elderly people, meta-analysis

## Abstract

**Objective:**

Sarcopenia is a typical age-related disorder characterized by loss of muscle mass, strength, and physical function. Resistance training has a noticeable effect on sarcopenia, but there is no consensus on whether nutritional supplements can boost this effect. We conducted a meta-analysis of relevant literature to investigate the therapeutic effect of resistance training combined with nutrition intervention on sarcopenia compared with resistance training alone.

**Methods:**

Cochrane Library, PubMed, Web of Science, Embase, Sinomed, CNKI, VIP, and Wanfang Data were searched for relevant studies on resistance training combined with nutritional intervention for aging adults with sarcopenia. The retrieval period ranged from the inception of the databases to May 24, 2022. Literature screening and information extraction were performed by two researchers. The Physiotherapy Evidence Database (PEDro) scale was adopted for literature quality evaluation and Stata 15.0 software for analysis.

**Results:**

Twelve clinical trials were included, involving 713 older adults diagnosed with sarcopenia, of whom 361 were assigned to the experimental group and 352 to the control group. Compared with the control group, grip strength of the experimental group was substantially elevated [WMD = 1.87, 95% CI (0.01, 3.74), *P* = 0.049]. Subgroup analysis demonstrated that vitamin D and protein increased grip strength and gait speed. There were no significant improvement in grip strength and gait speed in the protein and vitamin D free subgroup.

**Conclusions:**

This meta-analysis demonstrated that resistance training combined with additional nutritional supplementation, especially compound nutritional supplements that included protein and vitamin D, might further enhance grip strength rather than muscle mass in older adults with sarcopenia.

**Systematic review registration:**

https://www.crd.york.ac.uk/PROSPERO/, identifier: CRD42022346734.

## Background

Sarcopenia is recognized as a complex syndrome that tends to occur in older adults. It is a phenomenon of aging accompanied by progressive and systemic skeletal muscle decline, and it has been defined as loss of muscle mass, muscle strength, and physical function (1). Hereditary factors, lack of exercise, malnutrition, increased inflammation, age-related declines in hormone concentrations, and low vitamin D levels are currently regarded as risk factors for sarcopenia (2, 3). Estimated morbidity in both males and females over 60 years is 10% (4). Sarcopenia causes frailty, handicap, reduced quality of life, and even death in older adults (5).

Muscle mass and strength decline with age after middle age, and the development and progression of age-related primary sarcopenia may involve protein synthesis, proteolysis, muscle fat content, and neuromuscular integrity (6). The pathogenesis may be multifactorial. First, aging causes the disfunction of satellite cells, which play an important role in muscle regeneration, leading to a decline in the regeneration capacity and number of muscle fibers (7, 8). Various myocyte growth factors can also regulate the function of satellite cells to increase or reduce muscle fibers (9). Second, the activity of the IGF-1/PI3K/mTOR system, which plays an important role in muscle hypertrophy, decreases with aging (10). Furthermore, amyotrophy-related factors in skeletal muscles increase with aging (11), resulting in less muscle protein synthesis (MPS) and more muscle protein breakdown (MPB). Third, chronic inflammation induced by various factors in aging compromises muscle strength and function by enhancing the infiltration of macrophage into skeletal muscle, reducing muscle mass, and increasing the accumulation of ectopic fat (12). Fourth, the renin-angiotensin system (RAS) promotes muscle protein degradation through: (i) direct oxidative stress via angiotensin II type 1 receptors; (ii) decreased anabolic hormones via indirect induction; (iii) induction of proinflammatory cytokines; and (iv) enhanced muscle protein degradation via increased myostatin (9). Fifth, some relevant studies have reported that sex hormones (e. g., serum testosterone and estrogen) decreasing with age may be associated with sarcopenia (13, 14). Sixth, excessive mitochondrial reactive oxygen species (ROS) accumulate in skeletal muscle cells, and the accumulation of single-strand breaks in the telomeric region may accelerate telomere erosion, leading to cellular senescence (15). Besides, the generation of ROS causes inflammatory markers to overexpress and induces alterations in transcription factors and gene expression, which can affect protein equilibrium (16) and reduce muscle protein.

Skeletal muscle mass is determined by a complex balance between MPS and MPB, and resistance exercise is known as an effective stimulus to MPS promotion (17), and a recognized non-drug therapy that has produced satisfactory effects on the prevention and treatment of sarcopenia (18–22). However, whether nutritional supplementation can enhance the effect of resistance exercise remains uncertain. Although recommended by both the European Working Group on Sarcopenia in Older People (EWGSOP) and the Asian Working Group for Sarcopenia (AWGS) (1, 23), the “International Conference on Sarcopenia and Frailty Research” (ICFSR) indicated a low level of evidence (24). Some studies have reported that exercise training combined with dietary supplements has increased benefits, but some other research has conflicting findings (25). Currently, there is no systematic review and meta-analysis on nutritional supplementation combined with resistance exercises in older sarcopenic individuals. Therefore, the present study aimed to compare the therapeutic effect of resistance training combined with nutrition intervention vs. resistance training alone on muscular strength, quality, and function in elderly patients with sarcopenia.

## Materials and methods

This meta-analysis was conducted according to the Cochrane Handbook for the Systematic Review of Interventions (http://training.cochrane.org/handbook) and the Preferred Reporting Items for Systematic Review and Meta-Analyses. PMID: 19622552 (26). This study protocol was approved on PROSPERO (ID: CRD42022346734).

### Type of the included studies

Interventional study.

### Inclusion and exclusion criteria

The inclusion criteria were as follows. Participants: (i) aged 60 or above; (ii) diagnosed with sarcopenia. Since there is no unified definition of sarcopenia, we accept different definitions of “sarcopenia” in different studies; and (iii) have no major physical disability and can perform activities freely to ensure that they can perform resistance training normally. Intervention: (i) The experimental group performed resistance exercise combined with nutritional intervention as the experimental design, and took no specific drugs; The control group only performed resistance exercise and they were given placebo or isocaloric products with the same diet habit as usual. (ii) Intervention time ≥ 8 weeks, all participants had twice resistance exercise per week, and each time was not <20 min. The experimental group received resistance exercise plus intensive nutritional supplementation once or twice per day. (iii) The resistance exercise was designed as a progressive full-body workout with resistance equipment such as resistance bands or fitness facilities. Nutritional supplements included but were not limited to, protein, ω-3 fatty acids, vitamin D, and other substances that may be beneficial for improving muscle mass and strength.

The exclusion criteria were as follows: (i) Research disease was not related to sarcopenia; (ii) Research data was not accessible. (iii) Publication types were single-arm studies, reviews, pathological studies, animal experiments, dissertations or conference papers, etc.

#### Outcome indicators

According to the definition of sarcopenia, grip strength and chair stand test were used to evaluate the muscle strength of upper and lower limbs; total lean mass, appendicular skeletal muscle mass (ASM), and ASM index were used to assess muscle mass; timed up & go (TUG) and gait speed were used to evaluate muscle performance. Besides, body mass, body mass index (BMI), and total fat mass were used as secondary outcome indicators to observe the therapeutic effects of the two intervention methods on the elderly with sarcopenia.

### Retrieval strategies

A computer search was performed in Cochrane Library, PubMed, Web of Science, Embase, CNKI, and Wanfang Data, for research on the application effect of resistance training combined with nutritional intervention against sarcopenia. The retrieval period was from the inception of the databases to May 24, 2022. English key search terms included “sarcopenia”, “sarcopenias”, “training, resistance”, “strength training”, “nutrition therapy”, and “therapy, nutrition”. The Chinese terms used for search included “肌少症”, “骨骼肌减少症”, “抗阻训练”, and “营养”. Detailed search strategies are available in Supplementary Table 1.

### Literature screening and data extraction

Two researchers (ZS and TP) were responsible for the literature screening strictly following inclusion and exclusion criteria, and Endnote software version X9 was employed to manage the literature. The retrieved literature was initially imported into the software and the repeated literature was removed. Eligible studies were preliminarily selected by titles or abstracts, of which the full texts were downloaded. After reading the full texts, the original studies that met the requirements of this systematic review were included. Literature information was extracted; cross-validation was performed; and measurement units were unified. If there was any discrepancy, a third researcher (ZZ) was consulted to assist in the final decision. The extracted information mainly included title, first author, year of publication, country, type of study, diagnostic criteria for sarcopenia, sample size and gender of the experimental group and control group, intervention method, intervention time, and outcome indicators.

### Quality assessment

Two researchers (ZS and TP) assessed the quality of the included studies using the Physiotherapy Evidence Database (PEDro) scale (27). The PEDro scale consists of 11 items, including eligibility criteria, random allocation, concealed allocation, similar measures between groups at baseline, instructor blinding, assessor blinding, participant blinding, more than 85% dropout rate, intention-to-treat analysis, statistical comparison between groups, and ≥1 key outcome estimated. Each item is worth 1 point, except for criterion number 1. A higher total score indicates better methodological quality. Hence, a study with a PEDro score of 6 or higher was rated as high quality (6–8:good; 9–10:excellent), whereas a study with a score of 5 or lower was considered low quality (4–5: acceptable; < 4: poor) (28).

### Statistical methods

Stata 15.0 software was adopted to perform statistical analysis of the included literature, such as the tests for heterogeneity, publication bias analysis, and sensitivity analysis. The extracted data in the study were all continuous variables with unified measurement units. Effect sizes were combined using mean difference (MD), and 95% confidence intervals (CIs) were calculated. Heterogeneity was assessed using the Q statistic and I^2^ tests. If *P* > 0.1 and I^2^ ≤ 50%, the heterogeneity between studies was acceptable, and a fixed effect model was adopted for meta-analysis. If *P* ≤ 0.1 or I^2^ > 50%, heterogeneity between studies was high, and a random effect model was employed for meta-analysis. Publication bias of the included studies was verified by running the “metabias” command, and a *P* < 0.05 were considered statistically significant.

## Results

### Literature search results

**Figure 1:**
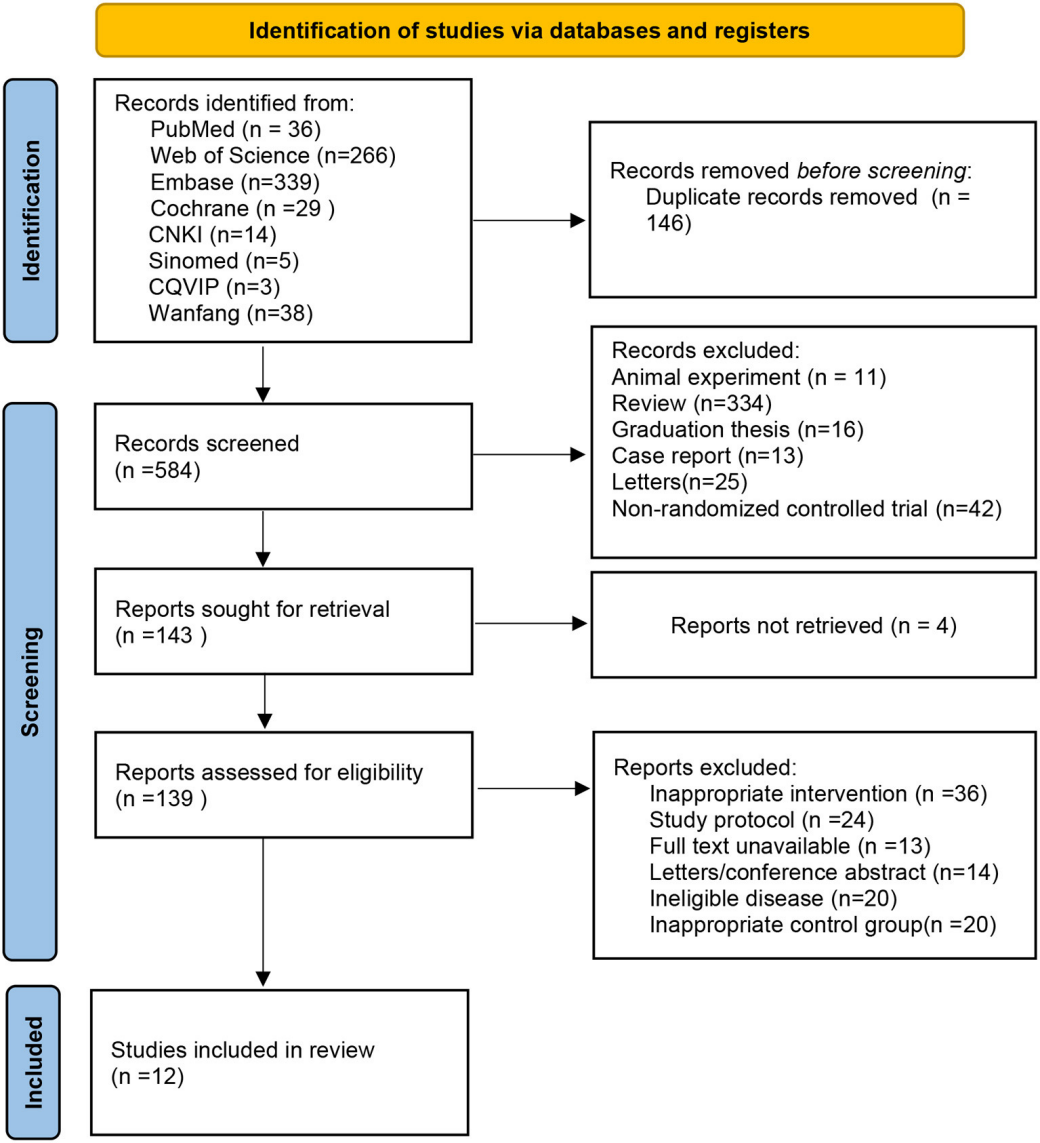
Literature screening flowchart and results.

A total of 730 articles were retrieved from the described databases, of which 29 articles were from Cochrane Library, 36 from PubMed, 266 from Web of Science, 339 from Embase, 5 from Sinomed, 14 from CNKI, 38 from Wanfang Data, and 3 from the VIP database. After the retrieved literature was imported into EndNote X9, 146 repeated literature were excluded. After reading the titles and abstracts, 441 irrelevant works of literature were removed. According to full-text reading, 131 unqualified works of literature were removed, Finally, 12 articles were included (29–40). The literature screening process is shown in Figure 1.

### Basic characteristics of the included literature

A total of 12 studies involving 713 older adults diagnosed with sarcopenia were included. The included studies were published in English. The basic characteristics of the included literature are shown in Table 1.

**Table 1:** The basic characteristics of the included literature.

**References**	**Country**	**PEDro score**	**Diagnosis criteria**	**Test group**		**Control group**	**Baseline dietary intake**		**Outcome indicators**
				**Resistance**	**Nutrition**		**Test group**	**Control group**	
Amasene et al. (29)	Spain	8	EWGSOP2	Resistance training, 1 h/time, 2 times/week, last for 12 weeks	3 g leucine, 20 g whey protein, 2 times/week, last for 12 weeks	placebo: maltodextrin			①②⑥⑦⑧⑨
Kim et al. (30)	Japan	7	into at least one inclusion criterion: (i) appendicular skeletal muscle mass/height^2^ < 6.42 kg/m^2^ and knee extension strength < 1.01 Nm/kg; (ii) appendicular skeletal muscle mass/height^2^ < 6.42 kg/m2^2^and usual walking speed < 1.10 m/s; (iii) BMI < 22 and knee extension strength < 1.10 Nm/kg; (iv) BMI < 22 and usual walking speed < 1.10 m/s.	Resistance training with resistance bands, 30 min/time, twice/week, last for 3 months	540 mg catechin, once/day, last for 3 months	diet as usual			①③⑤⑥
Kim et al. (31)	Japan	7	into at least one inclusion criterion: (i) appendicular skeletal muscle mass/height^2^ < 6.42 kg/m^2^ and knee extension strength < 1.01 Nm/kg; (ii) appendicular skeletal muscle mass/height^2^ < 6.42 kg/m2^2^and usual walking speed < 1.10 m/s; (iii) BMI < 22 and knee extension strength < 1.10 Nm/kg; (iv) BMI < 22 and usual walking speed < 1.10 m/s.	Progressive resistance training with resistance bands or ankle weights, 60 min/time, twice/week, last for 3 months	3 g amino acid, twice/day, last for 3 months	diet as usual			②⑥⑨
Forbeset al. (32)	China	7	AWGS	Strength training with dumbbells and sandbags for 20 min, 3 times/week, last for 12 weeks	30 g whey protein, 1000 IU vitamin D3, and 2 g ω-3 fatty acids, Once/day, last for 12 weeks	Standard personalized diet with an energy intake of 30 kcal/kg	High-quality protein intake (g):32.71 ± 11.81	High-quality protein intake (g):32.36 ± 10.74	①③④⑩
Maltais et al. (33)	Canada	8	Sarcopenia was determined as an appendicular lean mass index lower than 10.75 kg/m2	Free weights and resistance training with resistance equipment, 1 h/time, 3 times/week, last for 16 weeks	Milk powder mixed into 1% chocolate milk (375 ml, containing 13.53 g protein, 7 g EAA, 37.5 g carbohydrate, 3.8 g fat, 375 mg calcium, 270 calories). last for 16 weeks	Control: Rice emulsion as the control supplement	Protein intake (g/day):97 ± 79 Fat intake (g/day):137 ± 87 Energy intake (kcal/day):2326 ± 319 Carbohydrates intake (g/day):297 ± 50	Protein intake (g/day):92 ± 18 Fat intake (g/day):124 ± 88 Energy intake (kcal/day):2296 ± 345 Carbohydrates intake (g/day):277 ± 58	②④⑨⑩
Nabuco et al. (34)	Brazil	9	SO was defined as a body fat mass ≥35% combined with appendicular lean soft tissue (ALST) < 15.02 kg	Full body workout resistance training, 3 times/week, last for 12 weeks	35 g whey protein, 3 times/week, last for 12 weeks	placebo: maltodextrin	Protein intake (g/kg/day):0.93 ± 0.36 Lipids intake (g/day):51.8 ± 7.1 Energy intake (kcal/day):1539 ± 174 Carbohydrates intake (g/day):212.6 ± 29.3	Protein intake (g/kg/day):0.97 ± 0.28 Lipids intake (g/day):50.1 ± 7.3 Energy intake (kcal/day):1589 ± 154 Carbohydrates intake (g/day):224.4 ± 28.7	⑦⑩
Nilsson et al. (35)	Canada	9	EWGSOP	Home resistance training with full-body elastic bands, 3 times/week, last for 12 weeks	24 g whey, 16 g micelle casein, 3 g creatine, 1000 IU vitamin D3 and 2.46 g ω-3 fatty acids, Once/day, last for 12 weeks	placebo: Collagen and sunflower oil	Protein intake (g):88.7 ± 7 Fat intake (g):79.6 ± 6.7 Calories intake (kcal):2025 ± 131	Protein intake (g):81.2 ± 5.7 Fat intake (g):77.7 ± 5.9 Calories intake (kcal):1852 ± 107	①②③④⑤⑥⑦⑧
Shahar et al. (36)	Malaysia	5	assessed using bioimpedance analysis, skeletal muscle < 10.75 kg/m^2^ for men and 6.75 kg/m^2^ for women	Resistance training with resistance bands for 30 min, 2 times/week, last for 12 weeks	Soy protein drink for the protein supplement group, Powder protein intake of subjects was maintained up to 1.5 g/kg/d	diet as usual	Protein intake (g):55.63 ± 5.85 Fat intake (g):39.32 ± 10.91 Calories intake (kcal):1377.76 ± 153.49 Carbohydrate (Kcal):199.23 ± 22.25	Protein intake (g):54.89 ± 8.86 Fat intake (g):36.05 ± 4.72 Calories intake (kcal):1452.7 ± 192.91 Carbohydrate (Kcal):225.81 ± 35.55	①②⑥⑧⑨
Zdzieblik et al. (37)	Germany	7	EWGSOP	Resistance training with fitness facilities for 60 min, 3 times/week, last for 12 weeks	15 g collagen peptide, Once/day, last for 12 weeks	placebo: silicon dioxide			②⑧⑩
Zhu et al. (38)	China	8	AWGS	Resistance training with TheraBands and chairs for 20-30 min, 2 times/week, last for 12 weeks	17.22 g protein, 2.42 g β-hydroxyβ-methylbutyric acid, 260 IU vitamin D, and 0.58 g ω-3 fatty acids, Once/day, last for 12 weeks	diet as usual	Protein intake (g/day):76.3 ± 22.5 Energy intake (kcal/day):1598.7 ± 406.9	Protein intake (g/day):67.2 ± 18.1 Energy intake (kcal/day):1517.2 ± 349.4	①④⑥⑦
Rondanelli et al. (39)	Italy	8	Appendicular skeletal muscle mass/height^2^ < 7.26 kg/m^2^ for men and < 5.5 kg/m^2^ for women.	Upper and lower body strengthening with resistance bands per week, 20 min/time, 5 times/week, last for 12 weeks	Oral amino acids, whey protein, and vitamin D mixture	placebo: Containing isocaloric maltodextrin, the same taste, and appearance as the intervention products	Protein intake (g/day):54 ± 12 Fat intake (g/day):52 ± 9 Energy intake (kcal/day):1600 ± 215 Carbohydrates intake (g/day):214 ± 3	Protein intake (g/day):59 ± 8 Fat intake (g/day):54 ± 12 Energy intake (kcal/day):1622 ± 350 Carbohydrates intake (g/day):225 ± 4	①②④⑧⑨⑩
Rondanelli et al. (40)	Italy	9	EWGSOP	Loading exercises, Leg stretches and hip bends with resistance bands per week, 20-30 min/time, 5 times/week last for 8 weeks	Calorie formula containing 40 g powder (vanilla or strawberry flavor), 150 kcal, 20 g whey protein, 2.8 g leucine, 9 g carbohydrates, 3 g fat, 800 IU vitamin D, vitamin, a mixture of minerals (calcium, 500 mg) and fiber	with 40 g flavoring powder for maltodextrin (vanilla or strawberry flavor)	Protein intake (g/day):42.9 ± 11.7 Energy intake (kcal/day):1095.6 ± 179.6	Protein intake (g/day):41.5 ± 7.1 Energy intake (kcal/day):1144.9 ± 154.2	①③④⑤⑦

The resistance training regime was the same for the both control group and the experimental group. ① grip strength (kg); ② total lean body mass (kg); ③ appendicular skeletal muscle mass (kg); ④ appendicular skeletal muscle index (kg/m^2^); ⑤ timed “up & ⑥ go” test (s); ⑦ gait speed (m/s); ⑧ chair stand test (s); ⑨ weight (kg); ⑩ body mass index (kg/m^2^); fat mass (kg).

Notably, we extracted participants' baseline nutrition absorbed from their daily diets. Four studies (29–31, 37) did not provide baseline dietary intake, but one reported mini nutritional assessment scores and nutritional status (29). However, the nutritional status of the participants at enrollment in the other three studies was unknown due to the lack of baseline data. If most of them had an insufficient nutritional intake, it would amplify the effect of nutritional supplementation.

Protein was reported in three studies (33, 34, 36) amino acid powder supplements in one study (31), collagen peptides in one study (37), catechins in one study (30), leucine and whey protein in one study (29), and mixed nutritional supplements containing vitamin D and proteins in five studies (32, 35, 38–40).

### Quality appraisal of the included literature

The PEDro scores are shown in Table 1, and the detailed scores are presented in Supplementary Table 2. The scores for study quality ranged from 5 to 9. One study (36) scored 5 because it was quasi-experimental intervention research, and the rest of the studies were all randomized controlled trials with scores >6, indicating high quality.

### Meta-analysis results

#### Muscle strength

Muscle strength was assessed by indicators of grip strength and chair stand test. The grip strength and chair stand test were combined using the weighted MD (WMD).

#### Grip strength

**Figure 2:**
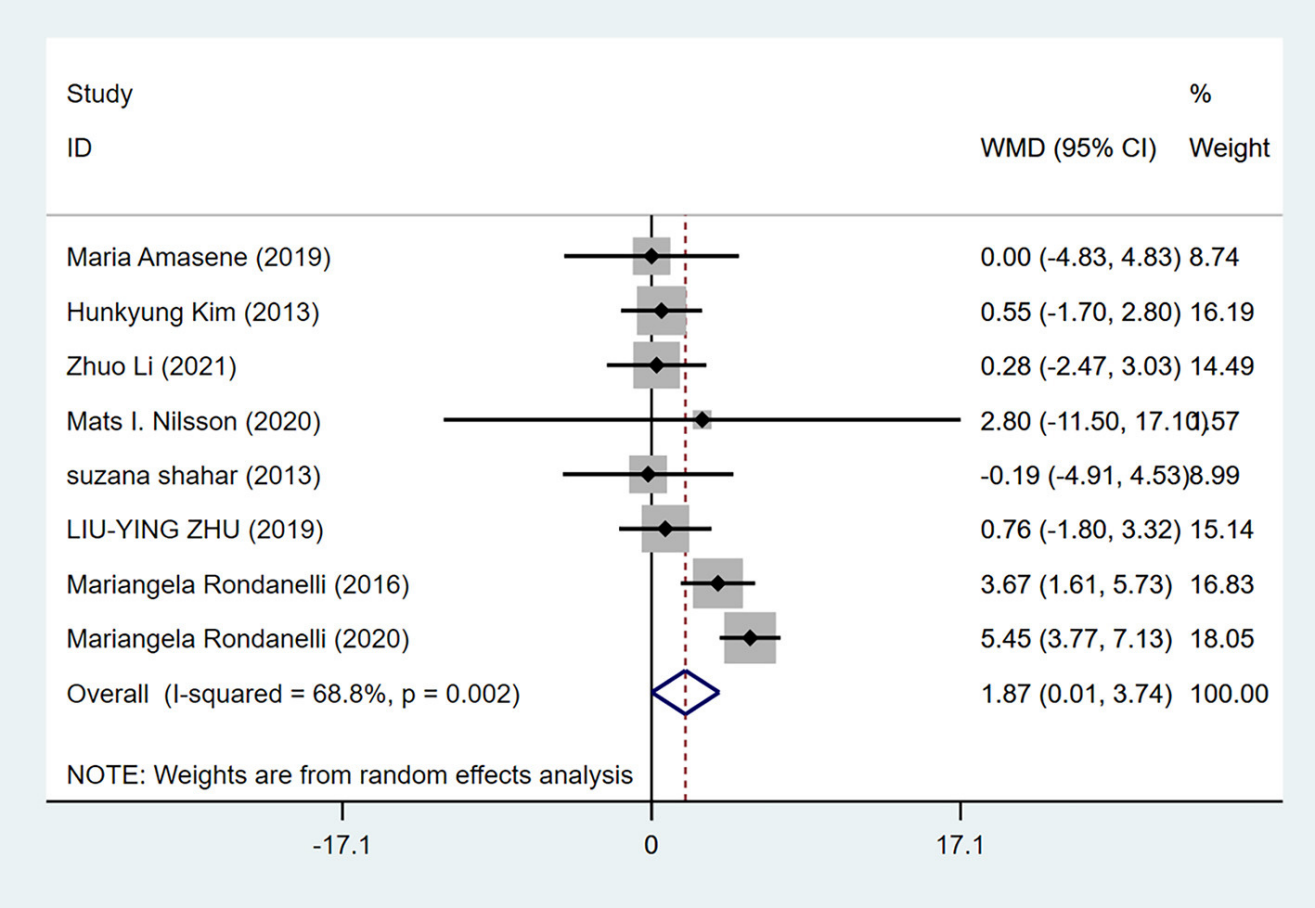
Forest plot for grip strength of resistance training combined with nutritional supplementation vs. resistance training alone. Overall estimates were obtained from forest plots of the meta-analysis using the random-effects model. Diamond icons and horizontal bars represent the overall estimate and 95% CI. WMD, weighted mean difference; CI, confidence interval.

Eight studies reported grip strength, with 280 cases in the experimental group and 266 cases in the control group. A random-effects model (I^2^=68.8%, *P* = 0.002) was used for meta-analysis, and the analysis results revealed that the difference in grip strength between the experimental group and the control group was statistically significant [WMD = 1.87, 95% CI (0.01, 3.74), *P* = 0.049], as shown in Figure 2.

#### Chair stand test

Five studies reported chair stand test, with 132 cases in the experimental group and 132 in the control group. A random-effects model (I^2^=86.6%, *P* = 0.000) was adopted for meta-analysis, and the results indicated no significant difference in chair stand test between the experimental group and the control group [WMD = −2.03, 95% CI (−5.82, 1.76), *P* = 0.295] as shown in Supplementary Figure 1.

#### Muscle mass

Muscle mass was assessed by total lean body mass, ASM, and ASM index. Since the measurement of related indicators used two different instruments, either dual-energy X-ray absorptiometry (DXA) or bioelectrical impedance analysis (BIA), we combined the results using standardized MD (SMD).

#### Lean body mass

Six studies reported lean body mass, with 229 cases in the experimental group and 214 cases in the control group. A fixed-effects model (I^2^ = 0.0%, *P* = 0.913) was employed to calculate the pooled effect sizes, and the results indicated no significant difference in lean body mass between the experimental group and the control group [SMD = 0.10, 95% CI (−0.14, 0.34), *P* = 0.422] as shown in Supplementary Figure 2.

#### Appendicular skeletal muscle mass

Five studies reported ASM, with 179 cases in the experimental group and 169 in the control group. A fixed-effects model (I^2^ = 0.0%, *P* = 0.928) was employed to calculate the pooled effect sizes, and the results indicated no significant difference in ASM between the experimental group and the control group [SMD = 0.15, 95% CI (−0.06, 0.36), *P* = 0.168] as presented in Supplementary Figure 3.

#### ASM index

Six studies reported the intervention effects of resistance training combined with nutritional supplementation and resistance training alone on old adults with sarcopenia, with 229 cases in the experimental group and 214 cases in the control group. A fixed-effects model (I^2^ = 0.0%, *P* = 0.807) was employed to calculate the pooled effect sizes, and the results indicated no significant difference in ASM index between the experimental group and the control group [SMD = 0.16, 95% CI (−0.03, 0.35), *P* = 0.096] as presented in Supplementary Figure 4.

#### Muscle performance

Muscle performance was assessed using TUG and gait speed. As studies with different gait speeds adopted different measures, we pooled the data using SMD.

#### TUG test

Four studies reported TUG, with 112 cases in the experimental group and 115 in the control group. A random-effects model (I^2^ = 81.4%, *P* = 0.001) was adopted to calculate the pooled effect sizes, and the results indicated no significant difference in TUG test between the experimental group and the control group [WMD = −0.81, 95% CI (−2.50, 0.88), *P* = 0.348] as shown in Supplementary Figure 5.

#### Gait speed

Seven studies gait speeds, with 197 cases in the experimental group and 204 cases in the control group. A fixed-effects model (I^2^ = 23.2%, *P* = 0.252) was employed to combine the effect sizes, and the results indicated no significant difference in gait speed between the experimental group and the control group [SMD = 0.17, 95% CI (−0.03, 0.36), *P* = 0.098] as presented in Supplementary Figure 6.

#### Secondary outcome indicators

For older adults with sarcopenia, there were no statistically significant differences in body mass, BMI, and fat mass between the experimental group and the control group (Supplementary Figures 7–9), as shown in Supplementary Table 3.

### Subgroup analysis

Subgroup analyses were performed according to the presence of protein and vitamin D in nutrients, and the results revealed an increase of grip strength in the protein and vitamin D subgroup [WMD = 2.72, 95% CI (0.38, 5.05), *P* = 0.022], whereas grip strength and gait speed were not statistically significant in the protein and vitamin D free subgroup [WMD = 0.35, 95% CI (−1.52, 2.22), *P* = 0.713] (Supplementary Figure 10). Gait speed was improved in the protein and vitamin D subgroup [SMD = 0.36, 95% CI (0.08, 0.63), *P* = 0.011], while there was no significant improvement in gait speed in the protein and vitamin D free subgroup [SMD = −0.04, 95% CI (−0.32, 0.25), *P* = 0.795], (Supplementary Figure 11). No significant differences were observed in skeletal muscle mass, TUG, and chair stand test between the two subgroups (Supplementary Figures 12–14).

Subgroup analysis was performed according to different diagnostic criteria. AWGS diagnostic criteria was used in two studies (32, 38), EWGSOP diagnostic criteria in three studies (35, 37, 40), and EWGSOP2 in one study (29). The findings suggested that the grip strength of the EWGSOP subgroup was significantly improved [WMD = 5.41, 95% CI (3.74, 7.09), *P* = 0.000], and there was no difference in the AWGS subgroup [WMD = 0.54, 95% CI (−1.34, 2.41), *P* = 0.574] (Supplementary Figure 15). EWGSOP2 trial findings revealed that protein supplementation did not further improve grip strength in sarcopenic patients after resistance training, and there was no difference between the EWGSOP and AWGS subgroups in skeletal muscle mass index (Supplementary Figure 16). There were insufficient data for subgroup analyses of the remaining indicators.

### Publication bias

Publication bias of the included studies was detected using Egger's test, and a *P* > 0.05 indicated the absence of publication bias. There was no publication bias in each index of the included literature (Table 2).

**Table 2:** Egger's test results of publication bias.

**Outcome indicators**	**Items**	**Effect size**	**Standard error**	**95% CI**	***t*-value**	***p***
Grip strength	Slope	5.041	1.875	[0.45, 9.63]	2.69	0.036
	Bias	−1.993	1.402	[−5.42, 1.43]	−1.42	0.205
Total lean body mass	Slope	0.257	0.159	[−0.18, 0.69]	1.62	0.182
	Bias	−0.581	0.531	[−2.05, 0.89]	−1.10	0.335
Appendicular skeletal muscle mass	Slope	0.288	0.141	[−0.17, 0.75]	1.98	0.143
	Bias	−0.619	0.607	[−2.55, 1.31]	−1.02	0.383
Appendicular skeletal muscle mass index	Slope	0.372	0.144	[−0.02, 0.77]	2.57	0.062
	Bias	−0.991	0.619	[−2.71, 0.72]	−1.60	0.185
Timed “up & go” test	Slope	1.399	1.900	[−6.77, 9.57]	0.74	0.538
	Bias	−2.833	2.961	[−15.57, 9.90]	−0.96	0.440
Gait speed	Slope	0.698	0.270	[0.003, 1.39]	2.58	0.049
	Bias	−2.121	1.013	[−4.72, 0.45]	−2.09	0.091
Chair stand test	Slope	1.034	2.844	[−8.01, 10.08]	0.36	0.740
	Bias	−1.705	2.422	[−9.41, 6.01]	−0.70	0.532
Weight	Slope	3.142	0.992	[0.38, 5.89]	3.17	0.034
	Bias	−0.537	0.289	[−1.34, 0.26]	−1.86	0.137
Body mass index	Slope	0.343	0.410	[−0.96, 1.64]	0.84	0.464
	Bias	−0.130	0.465	[−1.61, 1.35]	−0.28	0.797
Fat mass	Slope	−0.120	0.330	[−1.17, 0.92]	−0.37	0.739
	Bias	−0.15	1.293	[−4.31, 3.92]	−0.15	0.889

## Discussion

There are some debates over whether additional nutritional supplementation has better therapeutic effects for older sarcopenic individuals engaging in resistance exercise, and what nutritional supplements should be taken. As a result, the present study aimed to compare the beneficial effects of resistance training combined with nutritional supplementation vs. resistance training alone on older patients with sarcopenia, thereby providing comprehensive evidence for resource optimization and the improvement of the quality of life of older sarcopenia patients.

The current research analyzed 12 studies, including 11 randomized control trials and 1 quasi clinical trial, and the findings indicated that resistance training combined with extra nutritional supplements produced little effect on muscle mass, body mass, BMI, and fat mass in older patients with sarcopenia. After additional nutritional supplements were provided, especially complex supplements containing protein and vitamin D, grip strength and gait speed might be further improved in older adults with sarcopenia after resistance training. On the contrary, no improvement in lower limb strength assessed by the chair stand test was observed. According to a previous review, although some suggestive evidence revealed that a higher intake of nutritional supplements was associated with increased muscle strength, the finding was controversial with a lack of data on muscle strength (41). Hence, the present study can only conclude that additional nutritional supplements combined with resistance training can improve grip strength to some extent, whereas whether it can improve overall muscle strength needs to be further investigated.

Nutrients currently beneficial for sarcopenia management include protein, leucine, methyl β-hydroxyβ-butyrate, vitamin D, omega-3 fatty acids, and other antioxidants (42), all of which were reported in the included RCTs. Furthermore, one of the included RCTs focued on catechins. Most nutrients are not supplemented alone, and they are usually combined with other nutrients to form a compound nutrient. Protein was mostly reported in the included studies, with supplies ranging from a minimum of 13.53 g to a maximum of 35 g each time. Some studies have verified that older adults need protein intake of 1.6–1.8 g/kg/day to maintain muscle mass and function (43), which exceeds the recommended dietary allowance (RDA) of 0.8 g/kg/day (44). One of the possible reasons is that the anabolic resistance in older adults may inhibit the stimulating effects of strength exercise and protein intake on protein synthesis (45), so a higher protein intake is needed to maintain the synthesis. Notably, given the differences in participants' health statuses and dietary habits, for older people who have enough dietary protein intake each day, extra protein intake provides no more benefits, instead, its satiety properties may generate a “protein paradox,” which means that an excessive increase in protein intake may come at the expense of other important nutrients due to energy redistribution (46). A meta-analysis has indicated negative effects of whey protein supplementation at the same dose range (or even higher) as compared to control (47), and long-term high-protein diets may also affect colon health (48). The failure to improve muscle mass or performance may also be due to improper nutrition intake. In a case-control study, researchers used a specialized application program to provide health education and personalized nutrition and exercise guidance for older sarcopenia patients and observed improvements in muscle mass (49). Hence, simply increasing protein intake is not an optimal option, and daily dietary intake should be taken into account and evaluated carefully.

In terms of the duration of resistance exercise and nutritional supplementation, 3 months was reported in two studies, 12 weeks in eight studies, 8 weeks in one study, and 16 weeks in one study. The frequency of resistance training varied from 2 to 5 times per week. Taaffe et al. (50) have suggested that moderate-intensity training once or twice a week is sufficient to improve muscle mass (50). The frequency of nutritional supplementation ranged from 2 times a day to 3 times a week. Due to slower protein turnover in older adults, improvements in strength and physical performance are often observed before significant changes in muscle mass (51). This may be another reason why we did not observe a significant improvement in muscle mass. In this 16-week study (33), the researchers found that resistance training plus milk protein supplementation resulted in a significant increase in the lean body mass. Hence, appropriately extending the intervention duration without increasing frequency may improve muscle mass.

Protein and vitamin D were the most common combinations of compound nutrients, implying that the addition of vitamin D to a management regimen for sarcopenia may contribute to the recovery of functions of the patients (52). Subgroup analyses were performed for the studies containing both substances, and the findings indicated significant improvements in grip strength and gait speed in older patients with sarcopenia, which has important implications for clinical practice. However, the fact that this effect may be achieved due to other nutrients in the mixture can not be ignored. A previous meta-analysis indicated that vitamin D plus protein enhanced grip strength, but not gait speed, in patients with sarcopenia (53). Most of the RCTs included in that study did not perform resistance training, so the regime of protein and vitamin D supplements after resistance training may achieve better results.

Compared to previous studies, the present study is more targeted. Choi et al. (54) have published a study on the effects of resistance training combined with nutritional intervention vs. resistance training alone on healthy older adults, and they found that additional nutritional supplements did not improve muscle mass, strength, or performance in older adults engaging in resistance exercise. In contrast, the present study centered on older individuals with sarcopenia and found different results. The reason for the difference may be that older patients with sarcopenia were in poorer health and could gain more benefits from nutritional supplements. The meta-analysis published by Luo et al. (55) investigated the effect of nutritional supplements combined with exercise on elderly individuals with sarcopenia, while the present study focused on resistance exercise to minimize the impact of different forms of exercise on the study population. Furthermore, we included more high-quality original studies than previous studies.

An additional strength of this study is that it is the first meta-analysis comparing the effects of resistance training combined with nutritional supplementation vs. resistance training alone on elderly patients with sarcopenia. Besides, we performed a subgroup analysis according to nutritional combinations containing protein and vitamin D as well as different diagnostic criteria to conclude more meaningful results. To explore whether additional nutritional supplementation would improve the therapeutic effect of resistance training on older adults with sarcopenia, muscle strength, muscle mass, and muscle performance were assessed using multiple indicators.

The current study also has several limitations. First, the current study covers several types of nutrients, while the effect of a specific nutrient is not apparent. The reason is that, apart from protein-related studies, there are relatively few trials conducted to elucidate the effect of other kinds of nutrients. Subsequent trials focusing on a specific nutrient are necessary. Second, the improved effects on grip strength and gait speed cannot exclude the influence of additional nutrients. Thirdly, due to the lack of an international unified standard for the diagnosis of sarcopenia, the diagnostic criteria in half of the included studies were developed by their researchers themselves. Hence, the amount of muscle loss varied among the included populations. Fourth, the time, intensity, frequency, and nutrient supply of resistance training in the experiments were not consistent, which might have a certain impact on the research results. Finally, there are still few trials performed using the plan of several nutrients combined with resistance training for older patients with sarcopenia, and the number of participants in the study is also very limited. In one of the included studies, patients with sarcopenia were analyzed as a subgroup, and the number of these patients was only 7 (35), which might affect the reliability of the results.

The results of this study indicate that nutritional supplementation combined with resistance training does improve some aspects of muscle condition in older patients with sarcopenia, providing a basis for future nutritional supplementation for older sarcopenic patients. Furthermore, complex nutritional supplements rich in protein and vitamin D can be used for sarcopenic patients engaging in resistance training to further improve their muscle strength and performance.

## Conclusion

Combining nutritional supplementation with resistance training can improve grip strength in older patients with sarcopenia. Especially, complex nutritional supplements rich in protein and vitamin D can help the patients ameliorate their gait speed to some extent. Our results underpin the recommendation to combine nutritional supplementation with resistance training in older patients with sarcopenia. Reasonable and appropriate nutritional supplementation is advised to enhance the therapeutic effect of resistance training on older patients with sarcopenia. More high-quality, large-scale RCTs are desired to verify these findings.

## Data availability statement

The original contributions presented in the study are included in the article/Supplementary material, further inquiries can be directed to the corresponding author.

## Author contributions

ZS and ZZ contributed to conception and design of the study and organized the database. TP performed the statistical analysis. ZS wrote the first draft of the manuscript. TP and ZZ wrote sections of the manuscript. XT reviewed and edited the manuscript. YY made a critical review, commentary, and revision of the manuscript. All authors contributed to manuscript revision, read, and approved the submitted version.
