# Supplementary material for: The effects of nutritional supplementation on older sarcopenic individuals who engage in resistance training: a meta-analysis
Source: Front Nutr. 2023 Apr 25;10:1109789. doi: 10.3389/fnut.2023.1109789 (PMC10167299; doi:10.3389/fnut.2023.1109789)
Supplement: Supplementary file 1 [file Data_Sheet_1.docx]

**Supplementary Table 1** | Search strategy.

**1.Pubmed**

| Search number | Query | Results |
| --- | --- | --- |
| #1 | "Sarcopenia"[Mesh] | 7526 |
| #2 | (Sarcopenia[Title/Abstract]) OR (Sarcopenias[Title/Abstract]) | 12,923 |
| #3 | ("Sarcopenia"[Mesh]) OR ((Sarcopenia[Title/Abstract]) OR (Sarcopenias[Title/Abstract])) | 13,826 |
| #4 | "Resistance Training"[Mesh] | 11,205 |
| #5 | (((((((((((((((((((((((Resistance Training[Title/Abstract]) OR (Training, Resistance[Title/Abstract])) OR (Strength Training[Title/Abstract])) OR (Training, Strength[Title/Abstract])) OR (Weight-Lifting Strengthening Program[Title/Abstract])) OR (Strengthening Program, Weight-Lifting[Title/Abstract])) OR (Strengthening Programs, Weight-Lifting[Title/Abstract])) OR (Weight Lifting Strengthening Program[Title/Abstract])) OR (Weight-Lifting Strengthening Programs[Title/Abstract])) OR (Weight-Lifting Exercise Program[Title/Abstract])) OR (Exercise Program, Weight-Lifting[Title/Abstract])) OR (Exercise Programs, Weight-Lifting[Title/Abstract])) OR (Weight Lifting Exercise Program[Title/Abstract])) OR (Weight-Lifting Exercise Programs[Title/Abstract])) OR (Weight-Bearing Strengthening Program[Title/Abstract])) OR (Strengthening Program, Weight-Bearing[Title/Abstract])) OR (Strengthening Programs, Weight-Bearing[Title/Abstract])) OR (Weight Bearing Strengthening Program[Title/Abstract])) OR (Weight-Bearing Strengthening Programs[Title/Abstract])) OR (Weight-Bearing Exercise Program[Title/Abstract])) OR (Exercise Program, Weight-Bearing[Title/Abstract])) OR (Exercise Programs, Weight-Bearing[Title/Abstract])) OR (Weight Bearing Exercise Program[Title/Abstract])) OR (Weight-Bearing Exercise Programs[Title/Abstract]) | 15,792 |
| #6 | ("Resistance Training"[Mesh]) OR ((((((((((((((((((((((((Resistance Training[Title/Abstract]) OR (Training, Resistance[Title/Abstract])) OR (Strength Training[Title/Abstract])) OR (Training, Strength[Title/Abstract])) OR (Weight-Lifting Strengthening Program[Title/Abstract])) OR (Strengthening Program, Weight-Lifting[Title/Abstract])) OR (Strengthening Programs, Weight-Lifting[Title/Abstract])) OR (Weight Lifting Strengthening Program[Title/Abstract])) OR (Weight-Lifting Strengthening Programs[Title/Abstract])) OR (Weight-Lifting Exercise Program[Title/Abstract])) OR (Exercise Program, Weight-Lifting[Title/Abstract])) OR (Exercise Programs, Weight-Lifting[Title/Abstract])) OR (Weight Lifting Exercise Program[Title/Abstract])) OR (Weight-Lifting Exercise Programs[Title/Abstract])) OR (Weight-Bearing Strengthening Program[Title/Abstract])) OR (Strengthening Program, Weight-Bearing[Title/Abstract])) OR (Strengthening Programs, Weight-Bearing[Title/Abstract])) OR (Weight Bearing Strengthening Program[Title/Abstract])) OR (Weight-Bearing Strengthening Programs[Title/Abstract])) OR (Weight-Bearing Exercise Program[Title/Abstract])) OR (Exercise Program, Weight-Bearing[Title/Abstract])) OR (Exercise Programs, Weight-Bearing[Title/Abstract])) OR (Weight Bearing Exercise Program[Title/Abstract])) OR (Weight-Bearing Exercise Programs[Title/Abstract])) | 20,336 |
| #7 | "Nutrition Therapy"[Mesh] | 110,521 |
| #8 | ((((Nutrition Therapy[Title/Abstract]) OR (Therapy, Nutrition[Title/Abstract])) OR (Medical Nutrition Therapy[Title/Abstract])) OR (Nutrition Therapy, Medical[Title/Abstract])) OR (Therapy, Medical Nutrition[Title/Abstract]) | 2,351 |
| #9 | ("Nutrition Therapy"[Mesh]) OR (((((Nutrition Therapy[Title/Abstract]) OR (Therapy, Nutrition[Title/Abstract])) OR (Medical Nutrition Therapy[Title/Abstract])) OR (Nutrition Therapy, Medical[Title/Abstract])) OR (Therapy, Medical Nutrition[Title/Abstract])) | 111,610 |
| #10 | (("Nutrition Therapy"[Mesh]) OR (((((Nutrition Therapy[Title/Abstract]) OR (Therapy, Nutrition[Title/Abstract])) OR (Medical Nutrition Therapy[Title/Abstract])) OR (Nutrition Therapy, Medical[Title/Abstract])) OR (Therapy, Medical Nutrition[Title/Abstract]))) AND (("Resistance Training"[Mesh]) OR ((((((((((((((((((((((((Resistance Training[Title/Abstract]) OR (Training, Resistance[Title/Abstract])) OR (Strength Training[Title/Abstract])) OR (Training, Strength[Title/Abstract])) OR (Weight-Lifting Strengthening Program[Title/Abstract])) OR (Strengthening Program, Weight-Lifting[Title/Abstract])) OR (Strengthening Programs, Weight-Lifting[Title/Abstract])) OR (Weight Lifting Strengthening Program[Title/Abstract])) OR (Weight-Lifting Strengthening Programs[Title/Abstract])) OR (Weight-Lifting Exercise Program[Title/Abstract])) OR (Exercise Program, Weight-Lifting[Title/Abstract])) OR (Exercise Programs, Weight-Lifting[Title/Abstract])) OR (Weight Lifting Exercise Program[Title/Abstract])) OR (Weight-Lifting Exercise Programs[Title/Abstract])) OR (Weight-Bearing Strengthening Program[Title/Abstract])) OR (Strengthening Program, Weight-Bearing[Title/Abstract])) OR (Strengthening Programs, Weight-Bearing[Title/Abstract])) OR (Weight Bearing Strengthening Program[Title/Abstract])) OR (Weight-Bearing Strengthening Programs[Title/Abstract])) OR (Weight-Bearing Exercise Program[Title/Abstract])) OR (Exercise Program, Weight-Bearing[Title/Abstract])) OR (Exercise Programs, Weight-Bearing[Title/Abstract])) OR (Weight Bearing Exercise Program[Title/Abstract])) OR (Weight-Bearing Exercise Programs[Title/Abstract]))) | 304 |
| #11 | ((("Nutrition Therapy"[Mesh]) OR (((((Nutrition Therapy[Title/Abstract]) OR (Therapy, Nutrition[Title/Abstract])) OR (Medical Nutrition Therapy[Title/Abstract])) OR (Nutrition Therapy, Medical[Title/Abstract])) OR (Therapy, Medical Nutrition[Title/Abstract]))) AND (("Resistance Training"[Mesh]) OR ((((((((((((((((((((((((Resistance Training[Title/Abstract]) OR (Training, Resistance[Title/Abstract])) OR (Strength Training[Title/Abstract])) OR (Training, Strength[Title/Abstract])) OR (Weight-Lifting Strengthening Program[Title/Abstract])) OR (Strengthening Program, Weight-Lifting[Title/Abstract])) OR (Strengthening Programs, Weight-Lifting[Title/Abstract])) OR (Weight Lifting Strengthening Program[Title/Abstract])) OR (Weight-Lifting Strengthening Programs[Title/Abstract])) OR (Weight-Lifting Exercise Program[Title/Abstract])) OR (Exercise Program, Weight-Lifting[Title/Abstract])) OR (Exercise Programs, Weight-Lifting[Title/Abstract])) OR (Weight Lifting Exercise Program[Title/Abstract])) OR (Weight-Lifting Exercise Programs[Title/Abstract])) OR (Weight-Bearing Strengthening Program[Title/Abstract])) OR (Strengthening Program, Weight-Bearing[Title/Abstract])) OR (Strengthening Programs, Weight-Bearing[Title/Abstract])) OR (Weight Bearing Strengthening Program[Title/Abstract])) OR (Weight-Bearing Strengthening Programs[Title/Abstract])) OR (Weight-Bearing Exercise Program[Title/Abstract])) OR (Exercise Program, Weight-Bearing[Title/Abstract])) OR (Exercise Programs, Weight-Bearing[Title/Abstract])) OR (Weight Bearing Exercise Program[Title/Abstract])) OR (Weight-Bearing Exercise Programs[Title/Abstract])))) AND (("Sarcopenia"[Mesh]) OR ((Sarcopenia[Title/Abstract]) OR (Sarcopenias[Title/Abstract]))) | 36 |

**2.Cochrane**

| Search number | Query | Results |
| --- | --- | --- |
| #1 | MeSH descriptor: [Sarcopenia] explode all trees | 597 |
| #2 | (Sarcopenia):ti,ab,kw OR (Sarcopenias):ti,ab,kw | 1705 |
| #3 | MeSH descriptor: [Resistance Training] explode all trees | 4108 |
| #4 | (Resistance Training):ti,ab,kw OR (Training, Resistance):ti,ab,kw OR (Strength Training):ti,ab,kw OR (Training, Strength):ti,ab,kw OR (Weight-Lifting Strengthening Program):ti,ab,kw | 23122 |
| #5 | (Strengthening Program, Weight-Lifting):ti,ab,kw OR (Strengthening Programs, Weight-Lifting):ti,ab,kw OR (Weight Lifting Strengthening Program):ti,ab,kw OR (Weight-Lifting Strengthening Programs):ti,ab,kw OR (Weight-Lifting Exercise Program):ti,ab,kw | 266 |
| #6 | (Exercise Program, Weight-Lifting):ti,ab,kw OR (Exercise Programs, Weight-Lifting):ti,ab,kw OR (Weight Lifting Exercise Program):ti,ab,kw OR (Weight-Lifting Exercise Programs):ti,ab,kw OR (Weight-Bearing Strengthening Program):ti,ab,kw | 416 |
| #7 | (Strengthening Program, Weight-Bearing):ti,ab,kw OR (Strengthening Programs, Weight-Bearing):ti,ab,kw OR (Weight Bearing Strengthening Program):ti,ab,kw OR (Weight-Bearing Strengthening Programs):ti,ab,kw OR (Weight-Bearing Exercise Program):ti,ab,kw | 434 |
| #8 | (Exercise Program, Weight-Bearing):ti,ab,kw OR (Exercise Programs, Weight-Bearing):ti,ab,kw OR (Weight Bearing Exercise Program):ti,ab,kw OR (Weight-Bearing Exercise Programs):ti,ab,kw | 454 |
| #9 | MeSH descriptor: [Nutrition Therapy] explode all trees | 10290 |
| #10 | (Nutrition Therapy):ti,ab,kw OR (Therapy, Nutrition):ti,ab,kw OR (Medical Nutrition Therapy):ti,ab,kw OR (Nutrition Therapy, Medical):ti,ab,kw OR (Therapy, Medical Nutrition):ti,ab,kw | 8798 |
| #11 | #1 OR #2 | 1705 |
| #12 | #3 OR #4 OR #5 OR #6 OR #7 OR #8 | 23434 |
| #13 | #9 OR #10 | 16703 |
| #14 | #11 AND #12 AND #13 | 29 |

**3.Embase**

| Search number | Query | Results |
| --- | --- | --- |
| #1 | 'sarcopenia'/exp | 16038 |
| #2 | sarcopenia:ti,ab,kw OR sarcopenias:ti,ab,kw | 19850 |
| #3 | 'resistance training'/exp | 23695 |
| #4 | 'training, resistance':ti,ab,kw OR 'resistance training':ti,ab,kw OR 'training, strength':ti,ab,kw OR 'weight-lifting strengthening program':ti,ab,kw OR 'strengthening program, weight-lifting':ti,ab,kw OR 'strengthening programs, weight-lifting':ti,ab,kw OR 'weight lifting strengthening program':ti,ab,kw OR 'weight-lifting strengthening programs':ti,ab,kw OR 'weight-lifting exercise program':ti,ab,kw OR 'exercise program, weight-lifting':ti,ab,kw OR 'exercise programs, weight-lifting':ti,ab,kw OR 'weight lifting exercise program':ti,ab,kw OR 'weight-lifting exercise programs':ti,ab,kw OR 'weight-bearing strengthening program':ti,ab,kw OR 'strengthening program, weight-bearing':ti,ab,kw OR 'strengthening programs, weight-bearing':ti,ab,kw OR 'weight bearing strengthening program':ti,ab,kw OR 'weight-bearing strengthening programs':ti,ab,kw OR 'weight-bearing exercise program':ti,ab,kw OR 'exercise program, weight-bearing':ti,ab,kw OR 'exercise programs, weight-bearing':ti,ab,kw OR 'weight bearing exercise program':ti,ab,kw OR 'weight-bearing exercise programs':ti,ab,kw | 12603 |
| #5 | 'diet therapy'/exp | 401329 |
| #6 | 'nutrition therapy':ti,ab,kw OR 'therapy, nutrition':ti,ab,kw OR 'medical nutrition therapy':ti,ab,kw OR 'nutrition therapy, medical':ti,ab,kw OR 'therapy, medical nutrition':ti,ab,kw | 3549 |
| #7 | #1 OR #2 | 22080 |
| #8 | #3 OR #4 | 27282 |
| #9 | #5 OR #6 | 402425 |
| #10 | #7 AND #8 AND #9 | 339 |

**4.Web of science**

| Search number | Query | Results |
| --- | --- | --- |
| #1 | Sarcopenia (Topic) or Sarcopenias (Topic) | 23908 |
| #2 | Nutrition Therapy (Topic) or Therapy, Nutrition (Topic) or Medical Nutrition Therapy (Topic) or Nutrition Therapy, Medical (Topic) or Therapy, Medical Nutrition (Topic) 486320 | 486320 |
| #3 | Resistance Training (Topic) or Training, Resistance (Topic) or Strength Training (Topic) or Training, Strength (Topic) or Weight-Lifting Strengthening Program (Topic) or Strengthening Program, Weight-Lifting (Topic) or Strengthening Programs, Weight-Lifting (Topic) or Weight Lifting Strengthening Program (Topic) or Weight-Lifting Strengthening Programs (Topic) or Weight-Lifting Exercise Program (Topic) or Exercise Program, Weight-Lifting (Topic) or Exercise Programs, Weight-Lifting (Topic) or Weight Lifting Exercise Program (Topic) or Weight-Lifting Exercise Programs (Topic) or Weight-Bearing Strengthening Program (Topic) or Strengthening Program, Weight-Bearing (Topic) or Strengthening Programs, Weight-Bearing (Topic) or Weight Bearing Strengthening Program (Topic) or Weight-Bearing Strengthening Programs (Topic) or Weight-Bearing Exercise Program (Topic) or Exercise Program, Weight-Bearing (Topic) or Exercise Programs, Weight-Bearing (Topic) or Weight Bearing Exercise Program (Topic) or Weight-Bearing Exerc ise Programs (Topic) | 148313 |
| #4 | #1 AND #2 AND #3 | 266 |

**Supplementary Table 2 |** the detailed scores of PEDro scale.

| Study | Score | PEDro item number | | | | | | | | | | | |
| --- | --- | --- | --- | --- | --- | --- | --- | --- | --- | --- | --- | --- | --- |
|  |  | 1 | 2 | 3 | 4 | 5 | 6 | 7 | 8 | 9 | 10 | 11 |  |
| Maria Amasene 2019 | 8 | 1 | 1 | 0 | 1 | 1 | 0 | 1 | 1 | 1 | 1 | 1 |  |
| Hunkyung Kim 2013 | 7 | 1 | 1 | 1 | 1 | 0 | 0 | 1 | 1 | 0 | 1 | 1 |  |
| Hun Kyung Kim 2012 | 7 | 1 | 1 | 1 | 1 | 0 | 0 | 1 | 1 | 0 | 1 | 1 |  |
| Zhuo Li 2021 | 7 | 1 | 1 | 1 | 1 | 0 | 0 | 0 | 1 | 1 | 1 | 1 |  |
| Mathieu L. Maltais 2016 | 8 | 1 | 1 | 0 | 1 | 1 | 1 | 0 | 1 | 1 | 1 | 1 |  |
| Hellen C.G. Nabuco 2019 | 9 | 1 | 1 | 1 | 1 | 1 | 1 | 0 | 1 | 1 | 1 | 1 |  |
| Mats I. Nilsson 2020 | 9 | 1 | 1 | 1 | 1 | 1 | 1 | 0 | 1 | 1 | 1 | 1 |  |
| Suzana shahar 2013 | 5 | 1 | 0 | 0 | 1 | 0 | 0 | 0 | 1 | 1 | 1 | 1 |  |
| Denise Zdzieblik 2015 | 7 | 1 | 1 | 0 | 1 | 1 | 1 | 0 | 1 | 0 | 1 | 1 |  |
| LIU-YING ZHU 2019 | 8 | 1 | 1 | 1 | 1 | 1 | 0 | 0 | 1 | 1 | 1 | 1 |  |
| Mariangela Rondanelli 2016 | 8 | 1 | 1 | 0 | 1 | 1 | 1 | 0 | 1 | 1 | 1 | 1 |  |
| Mariangela Rondanelli 2020 | 9 | 1 | 1 | 1 | 1 | 1 | 1 | 0 | 1 | 1 | 1 | 1 |  |

Scale of item score:0, absent;1, present. The PEDro scale criteria are (1) eligibility criteria, (2) random allocation, (3) concealed allocation, (4) similarity at baseline on key measures, (5) subject blinding, (6) therapist blinding, (7) assessor blinding, (8) more than 85% follow-up of at least 1 key outcome, (9) intention-to-treat analysis, (10) between-group statistical comparison for at least one key outcome, and (11) point estimates and measures of variability provided for at least one key outcome. Item 1 is related to external validity and not used in the method score.

**Supplementary Table 3 |** Other outcome indicators.

| Outcome indicators | Cases of test group | Cases of control group | Statistical heterogeneity | WMD/SMD | 95%CI | P |
| --- | --- | --- | --- | --- | --- | --- |
| Weight | 193 | 186 | I²=0.0%, P=0.985 | WMD=1.47 | (-1.27, 4.29) | 0.294 |
| Body mass index | 141 | 145 | I²=0.0%, P=0.947 | WMD=0.24 | (-0.53, 1.01) | 0.540 |
| Fat mass | 164 | 148 | I²=0.0%, P=0.592 | SMD=-0.17 | (-0.39, 0.06) | 0.142 |


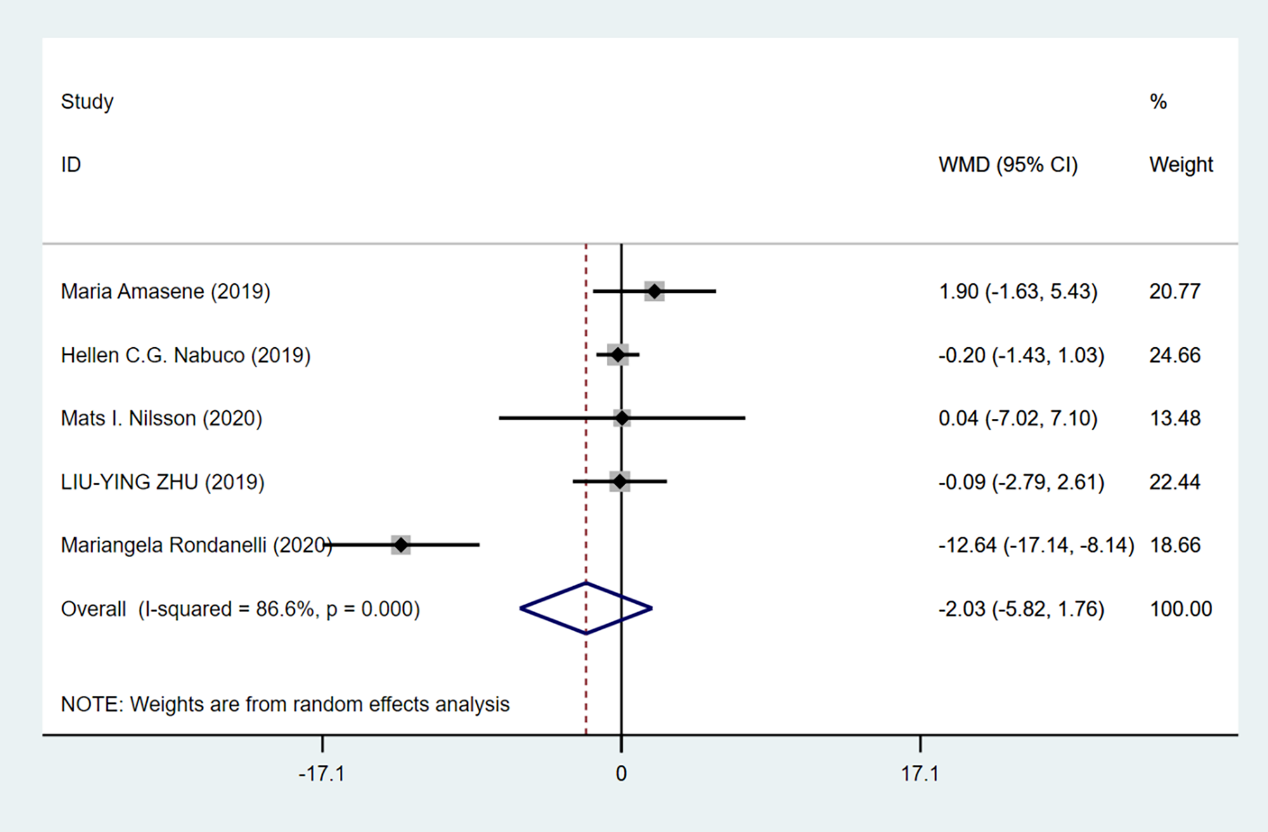


**Supplementary Figure 1** **|** Forest plot for chair stand test of resistance training combined with nutritional supplementation vs. resistance training alone. Overall estimates were obtained from forest plots of the meta-analysis using the random-effects model. Diamond icons and horizontal bars represent the overall estimate and 95% CI. WMD, weighted mean difference; CI, confidence interval.


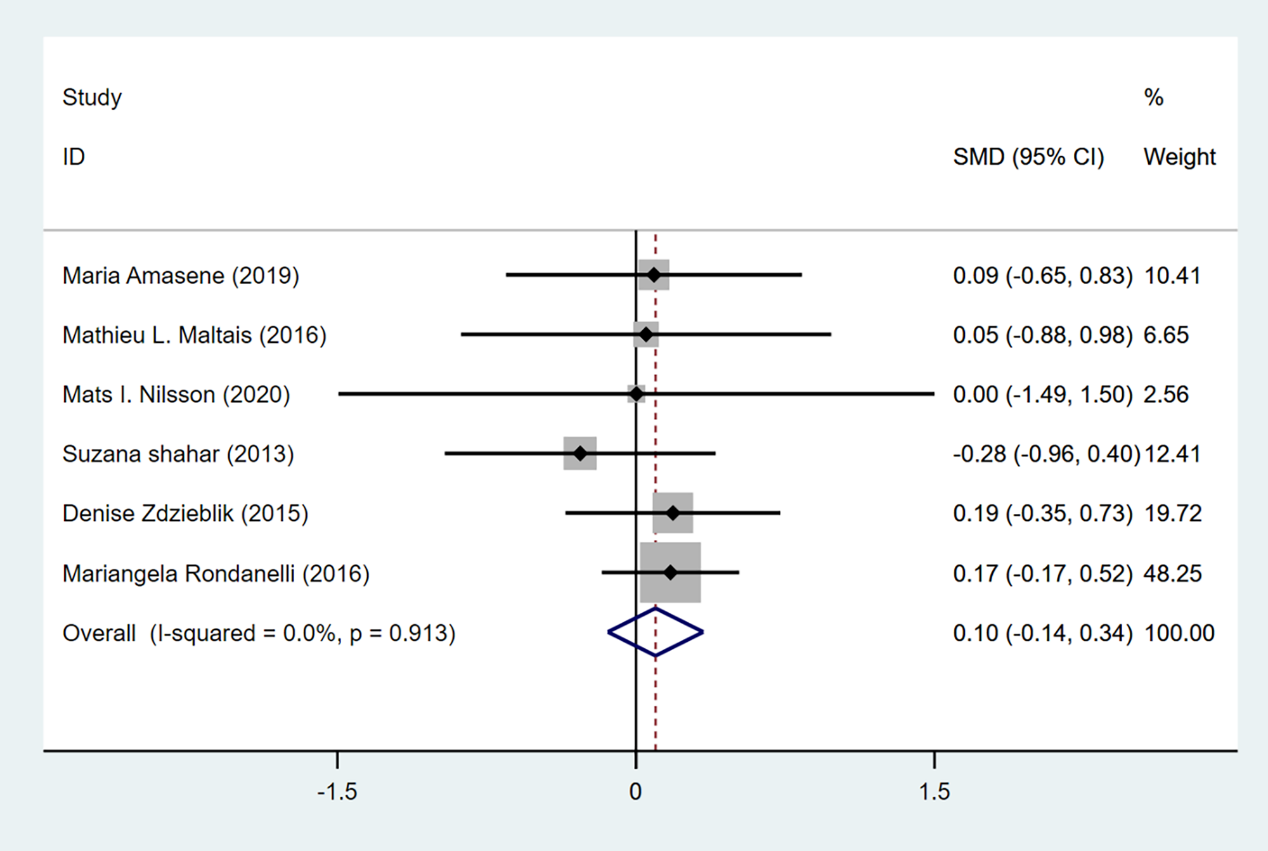


**Supplementary Figure 2** **|** Forest plot for lean body mass of resistance training combined with nutritional supplementation vs. resistance training alone. Overall estimates were obtained from forest plots of the meta-analysis using the fixed-effects model. Diamond icons and horizontal bars represent the overall estimate and 95% CI. SMD, standardized mean difference; CI, confidence interval.


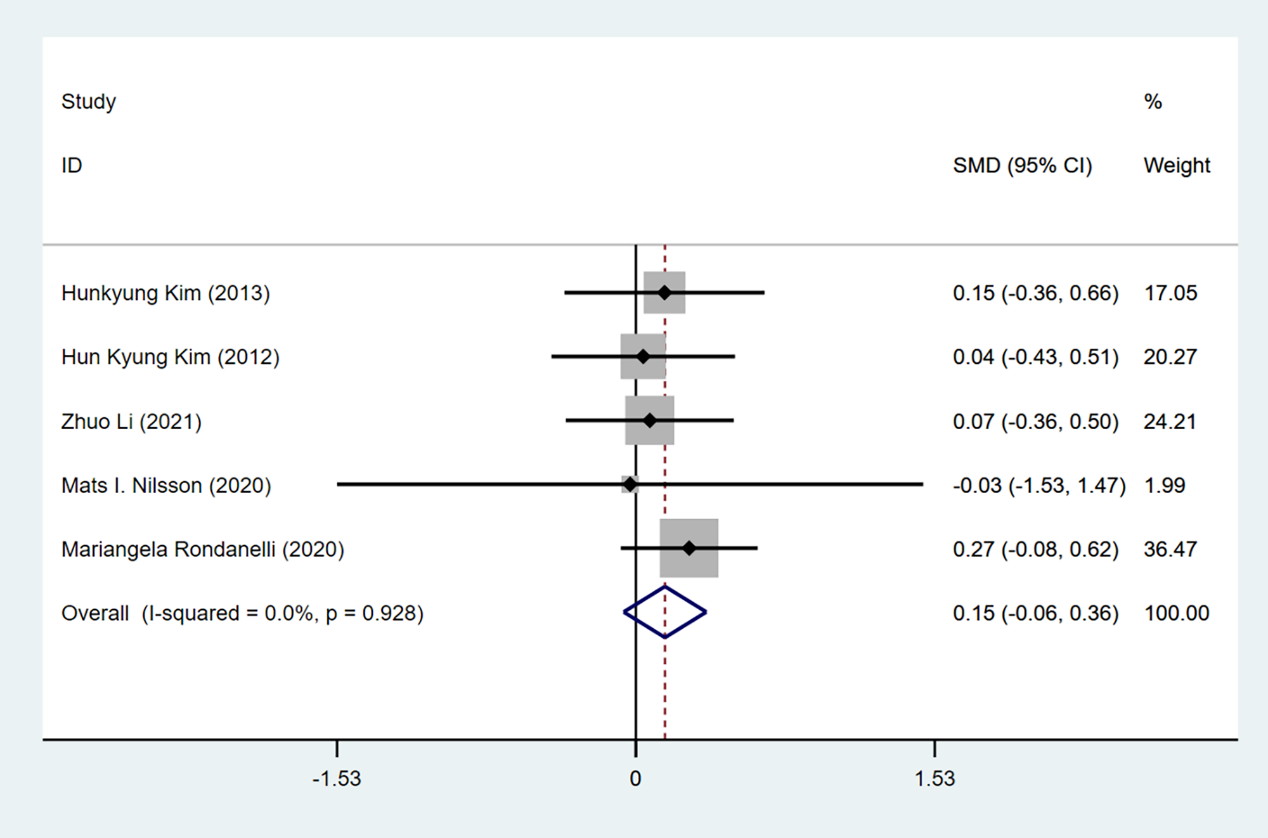


**Supplementary Figure 3** **|** Forest plot for appendicular skeletal muscle mass of resistance training combined with nutritional supplementation vs. resistance training alone. Overall estimates were obtained from forest plots of the meta-analysis using the fixed-effects model. Diamond icons and horizontal bars represent the overall estimate and 95% CI. SMD, standardized mean difference; CI, confidence interval.


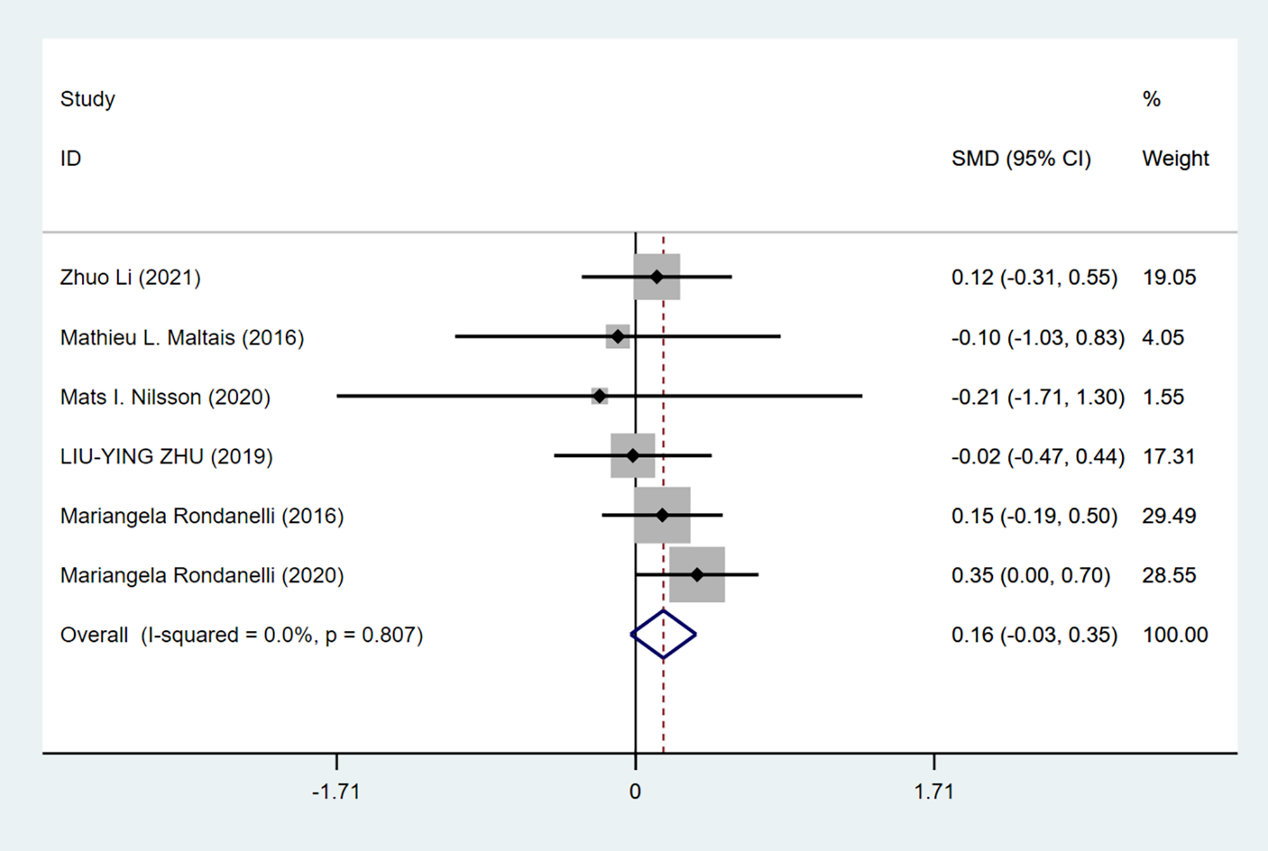


**Supplementary Figure 4** **|** Forest plot for appendicular skeletal muscle mass index of resistance training combined with nutritional supplementation vs. resistance training alone. Overall estimates were obtained from forest plots of the meta-analysis using the fixed-effects model. Diamond icons and horizontal bars represent the overall estimate and 95% CI. SMD, standardized mean difference; CI, confidence interval.


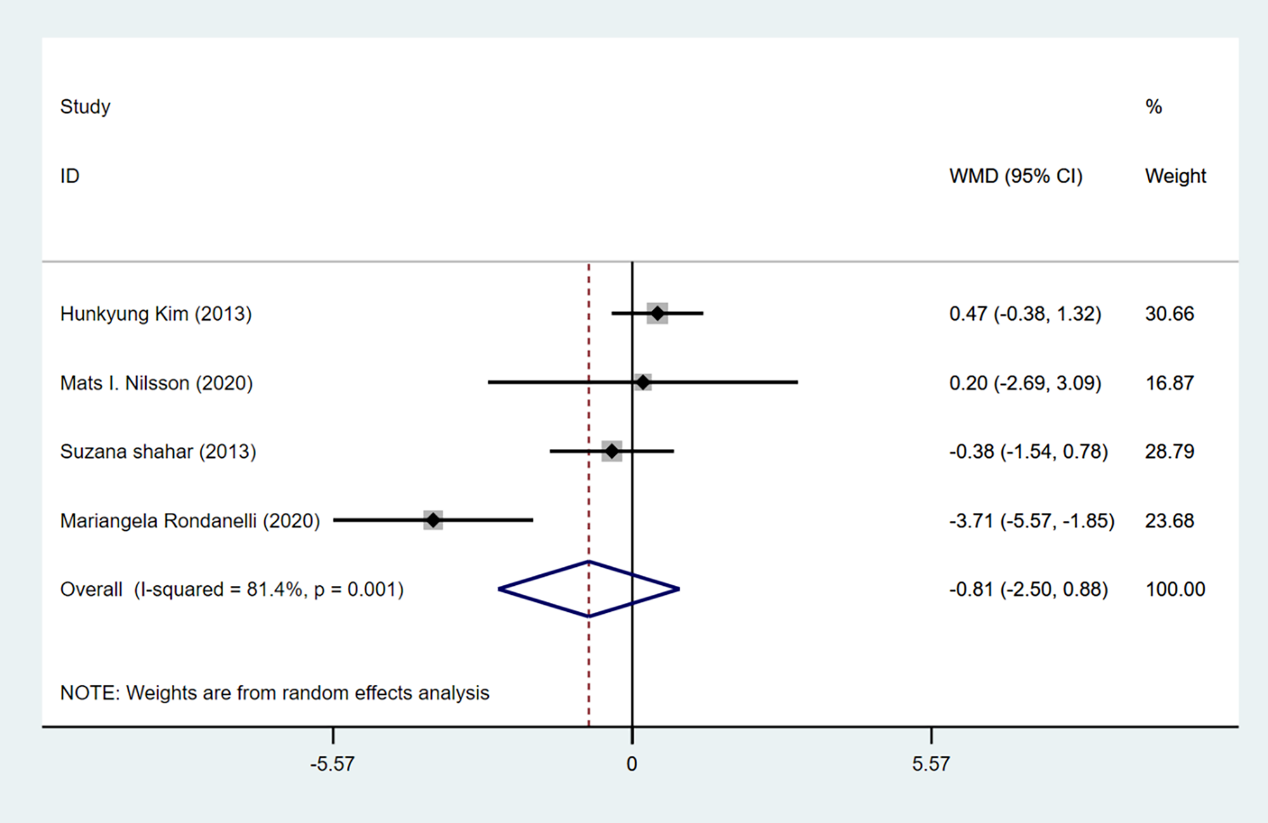


**Supplementary Figure 5** **|** Forest plot for TUG of resistance training combined with nutritional supplementation vs. resistance training alone. Overall estimates were obtained from forest plots of the meta-analysis using the random-effects model. Diamond icons and horizontal bars represent the overall estimate and 95% CI. WMD, weighted mean difference; CI, confidence interval.


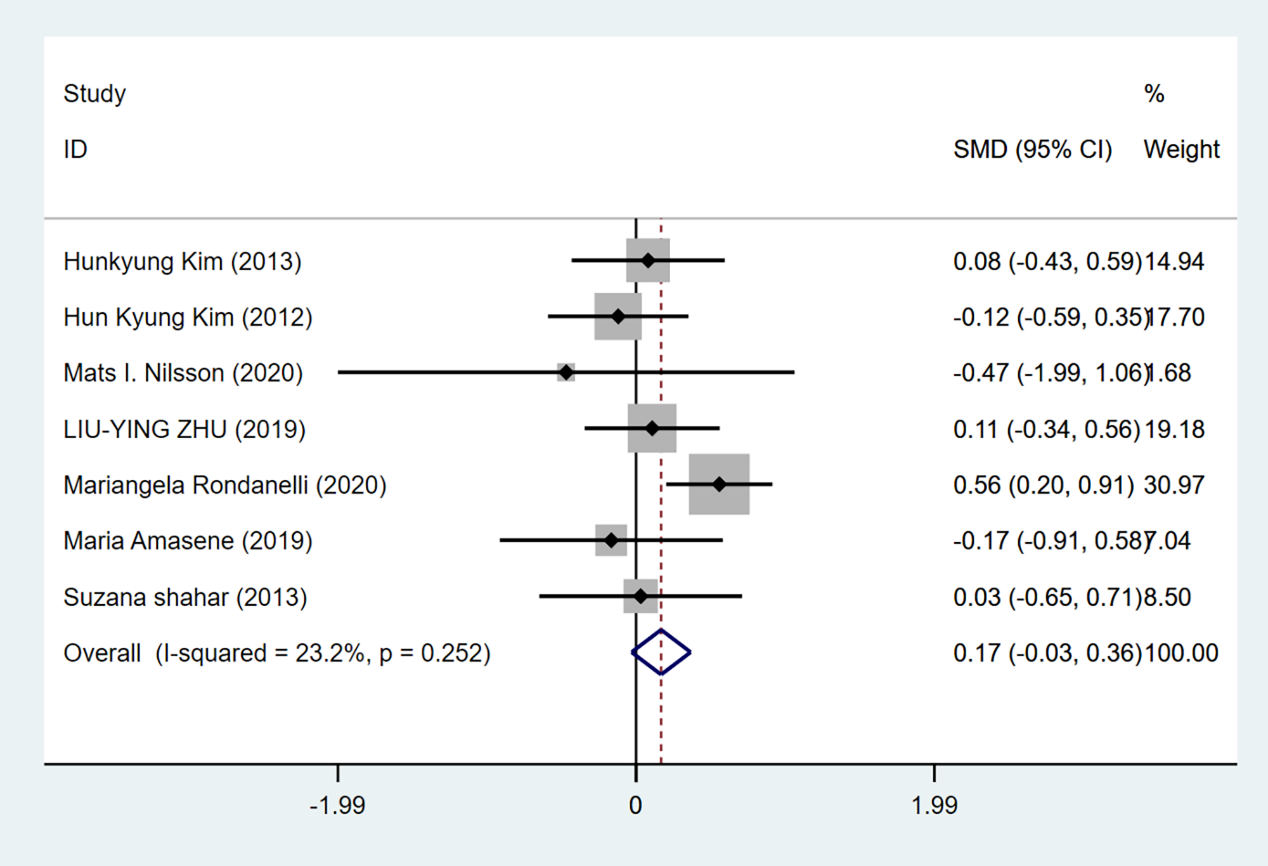


**Supplementary Figure 6** **|** Forest plot for gait speed of resistance training combined with nutritional supplementation vs. resistance training alone. Overall estimates were obtained from forest plots of the meta-analysis using the fixed-effects model. Diamond icons and horizontal bars represent the overall estimate and 95% CI. SMD, standardized mean difference; CI, confidence interval.


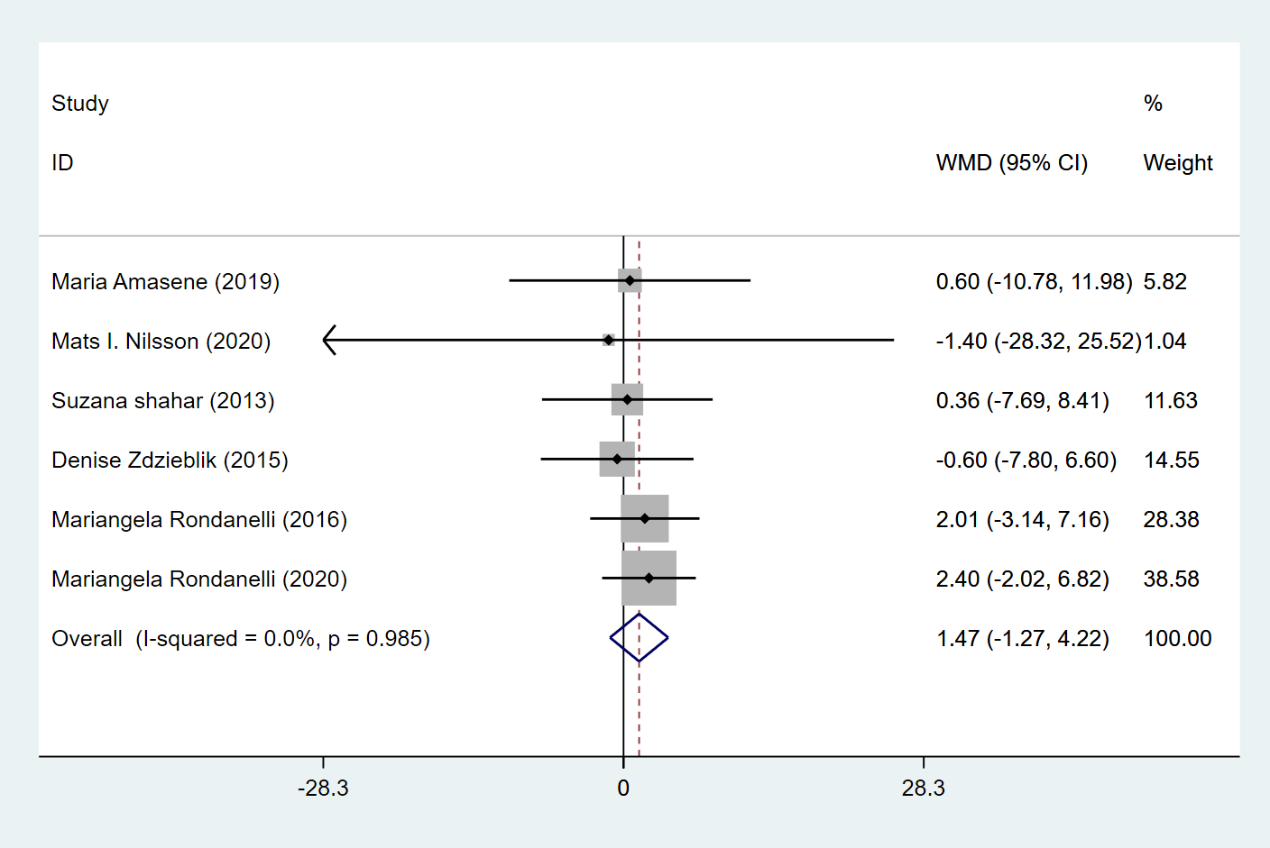


**Supplementary Figure 7** **|** Forest plot for body weight of resistance training combined with nutritional supplementation vs. resistance training alone. Overall estimates were obtained from forest plots of the meta-analysis using the fixed-effects model. Diamond icons and horizontal bars represent the overall estimate and 95% CI. WMD, weighted mean difference; CI, confidence interval.


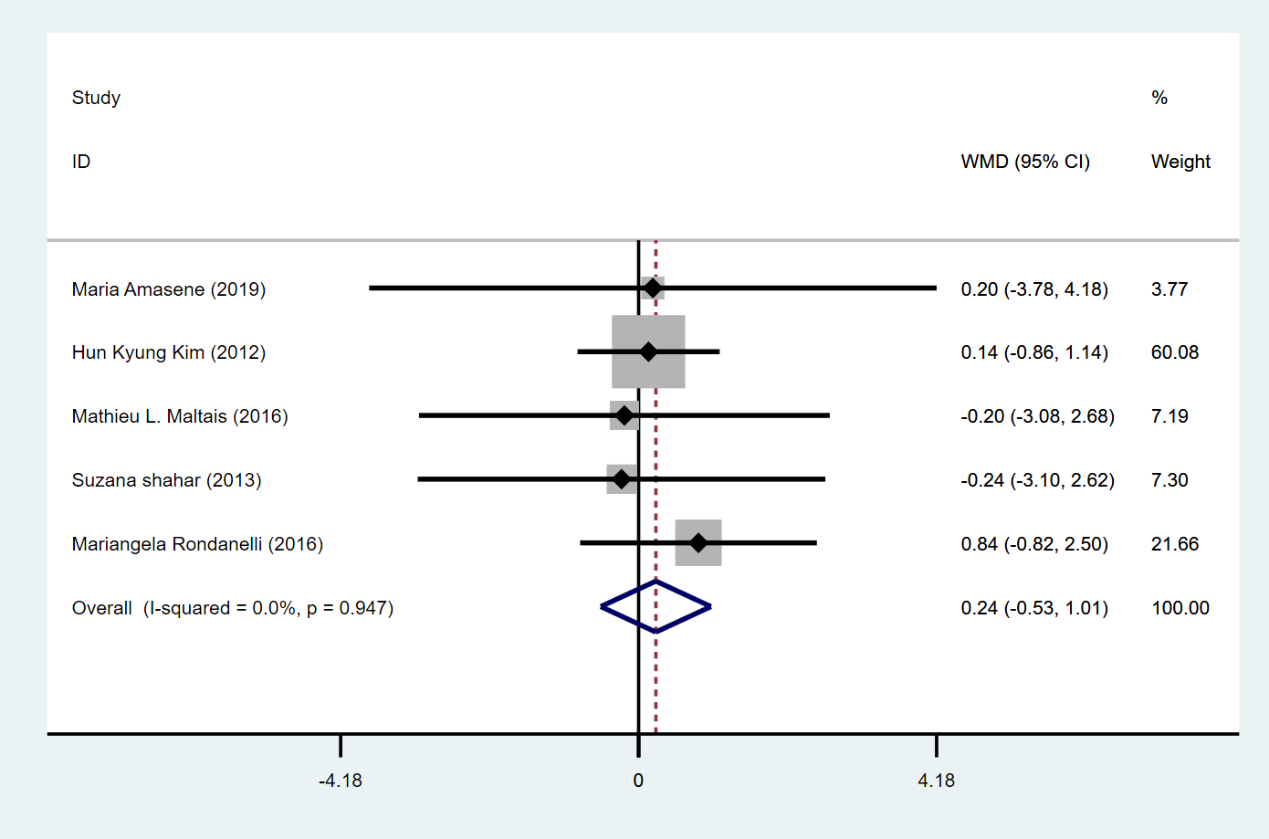


**Supplementary Figure 8 |** Forest plot for BMI of resistance training combined with nutritional supplementation vs. resistance training alone. Overall estimates were obtained from forest plots of the meta-analysis using the fixed-effects model. Diamond icons and horizontal bars represent the overall estimate and 95% CI. WMD, weighted mean difference; CI, confidence interval.


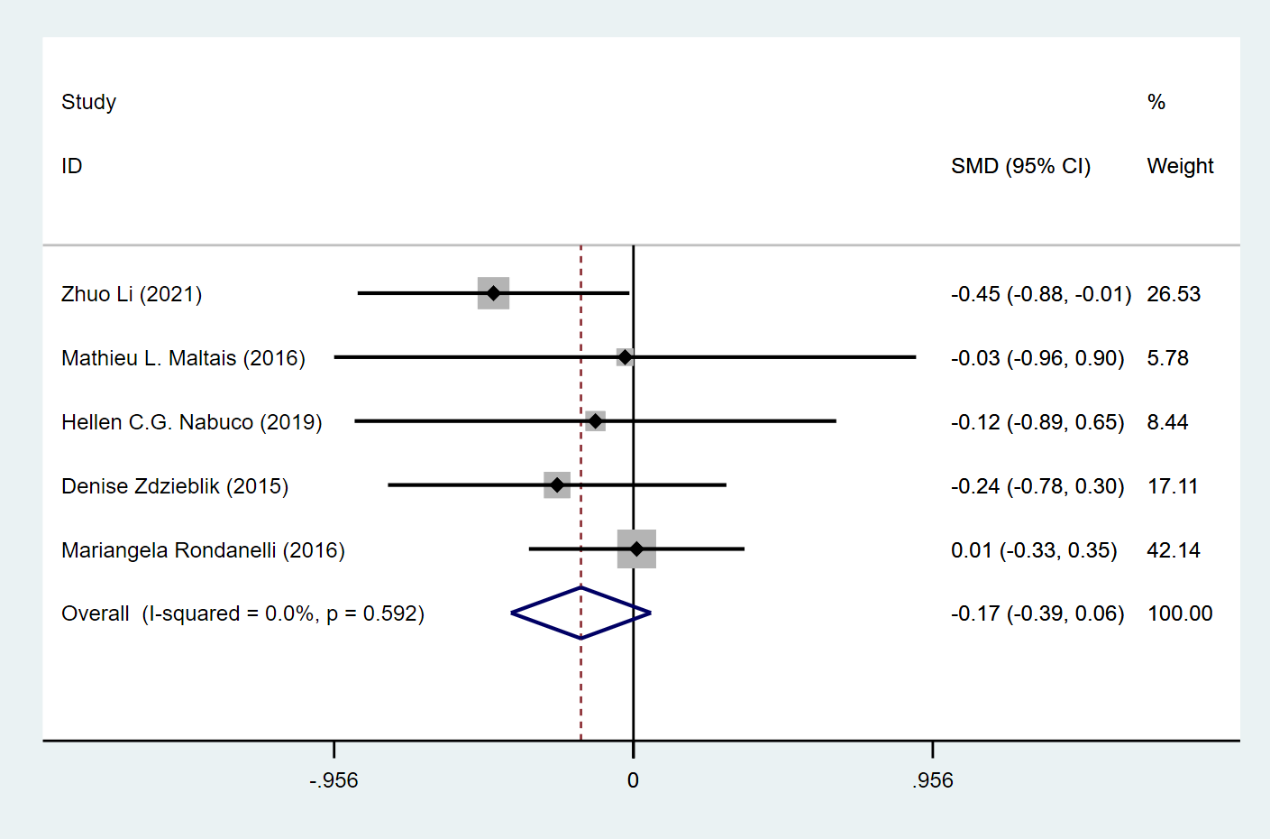


**Supplementary Figure 9** **|** Forest plot for fat mass of resistance training combined with nutritional supplementation vs. resistance training alone. Overall estimates were obtained from forest plots of the meta-analysis using the fixed-effects model. Diamond icons and horizontal bars represent the overall estimate and 95% CI. SMD, standardized mean difference; CI, confidence interval.


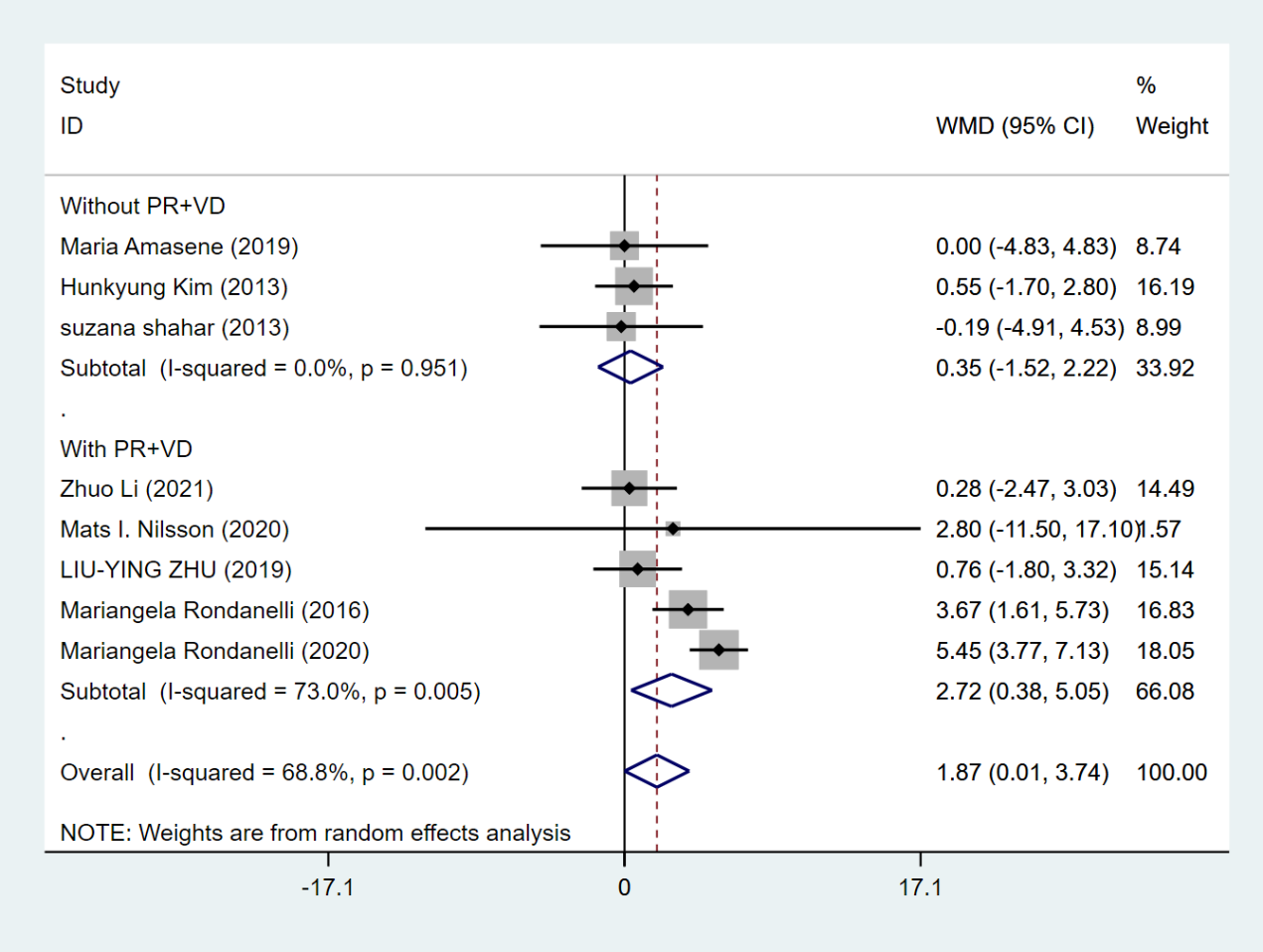


**Supplementary Figure 10** **|** Forest plot for grip strength of resistance training combined with nutritional supplementation vs. resistance training alone in the with or without PR+VD subgroup. Overall estimates were obtained from forest plots of the meta-analysis using the random-effects model. Diamond icons and horizontal bars represent the overall estimate and 95% CI. WMD, weighted mean difference; CI, confidence interval.


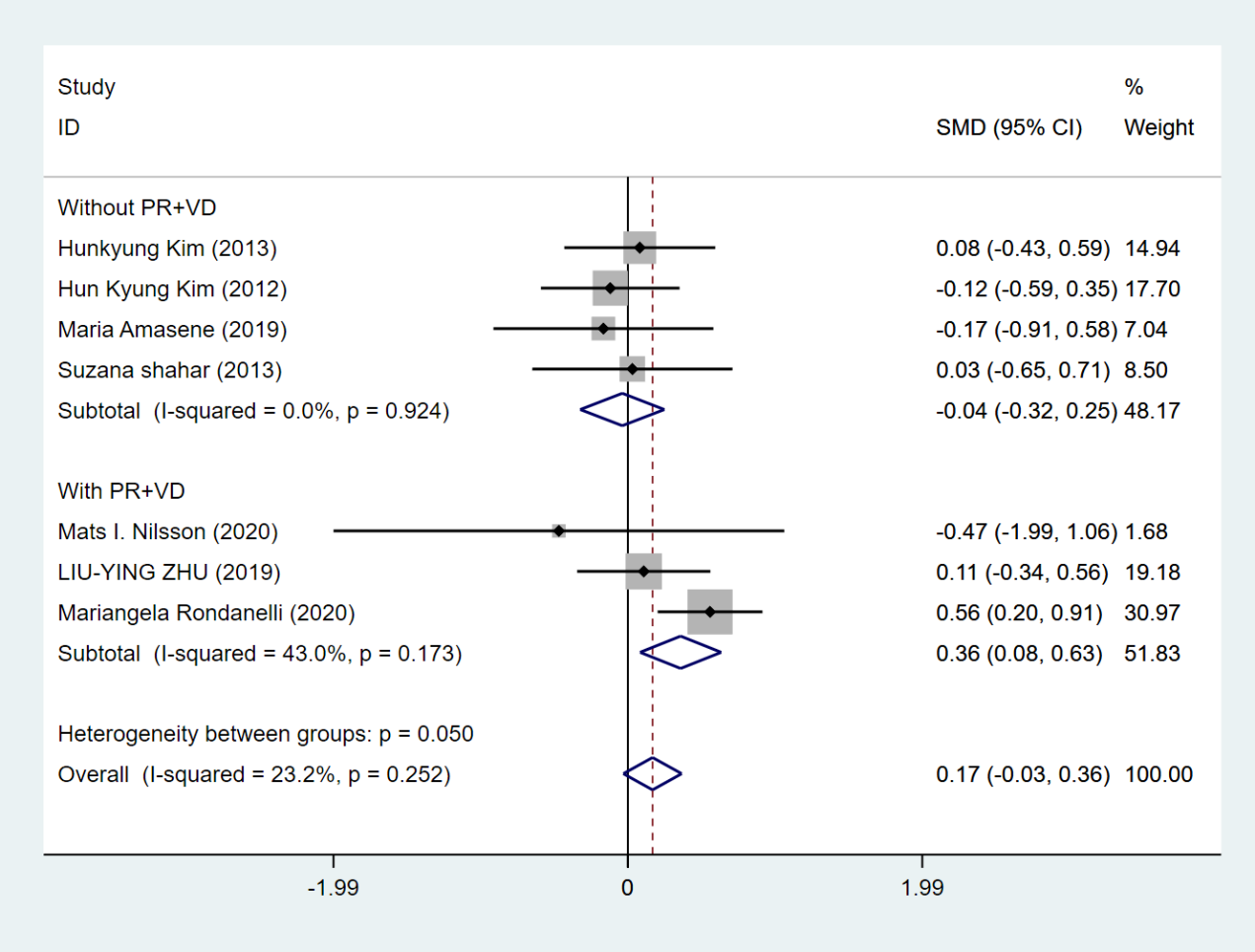


**Supplementary Figure 11** **|** Forest plot for gait speed of resistance training combined with nutritional supplementation vs. resistance training alone in the with or without PR+VD subgroup. Overall estimates were obtained from forest plots of the meta-analysis using the fixed-effects model. Diamond icons and horizontal bars represent the overall estimate and 95% CI. SMD, standardized mean difference; CI, confidence interval.


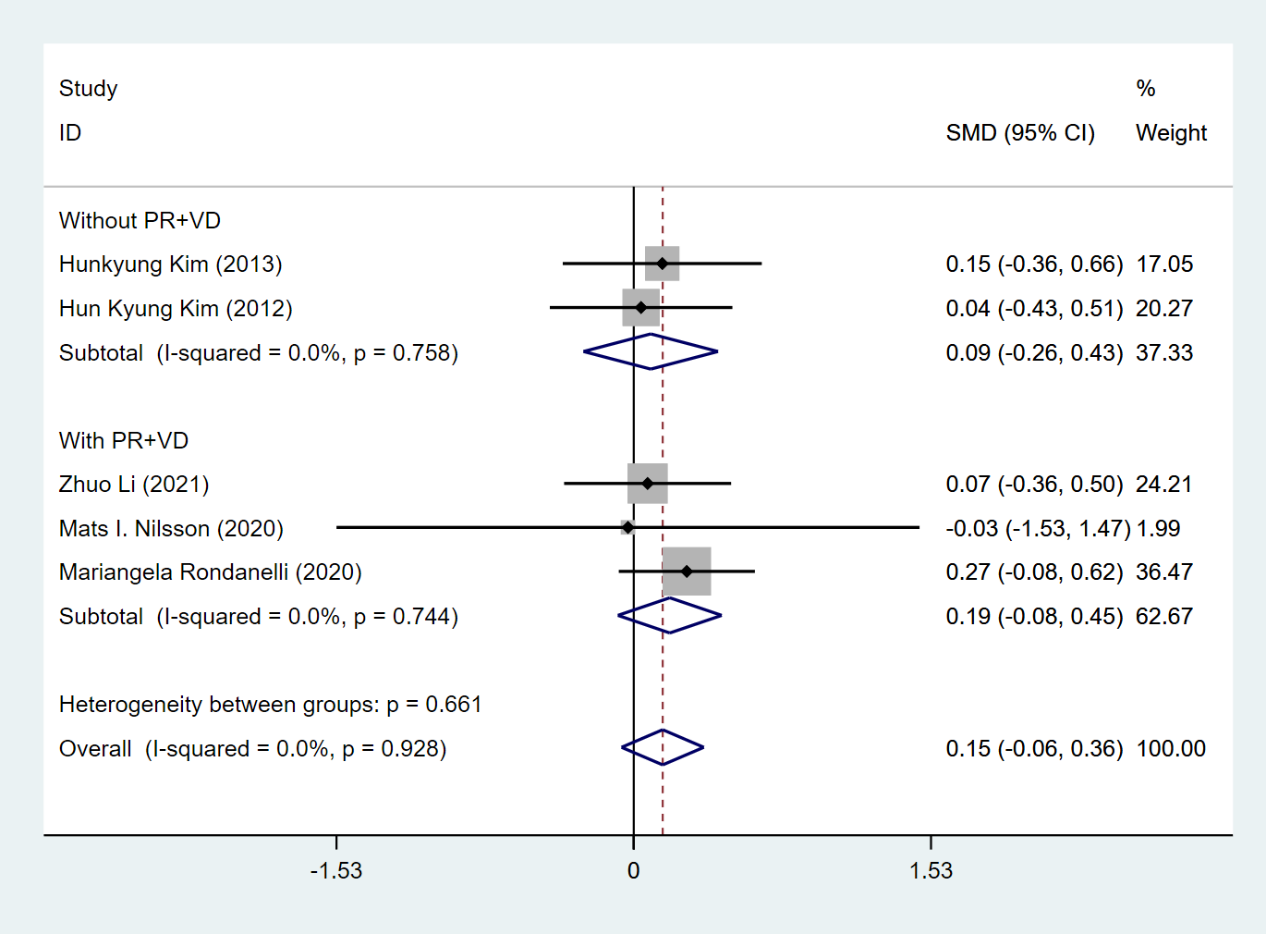


**Supplementary Figure 12** **|** ASM forest plot of resistance training combined with nutritional supplementation vs. resistance training alone in the with or without PR+VD subgroup. Overall estimates were obtained from forest plots of the meta-analysis using the fixed-effects model. Diamond icons and horizontal bars represent the overall estimate and 95% CI. SMD, standardized mean difference; CI, confidence interval.


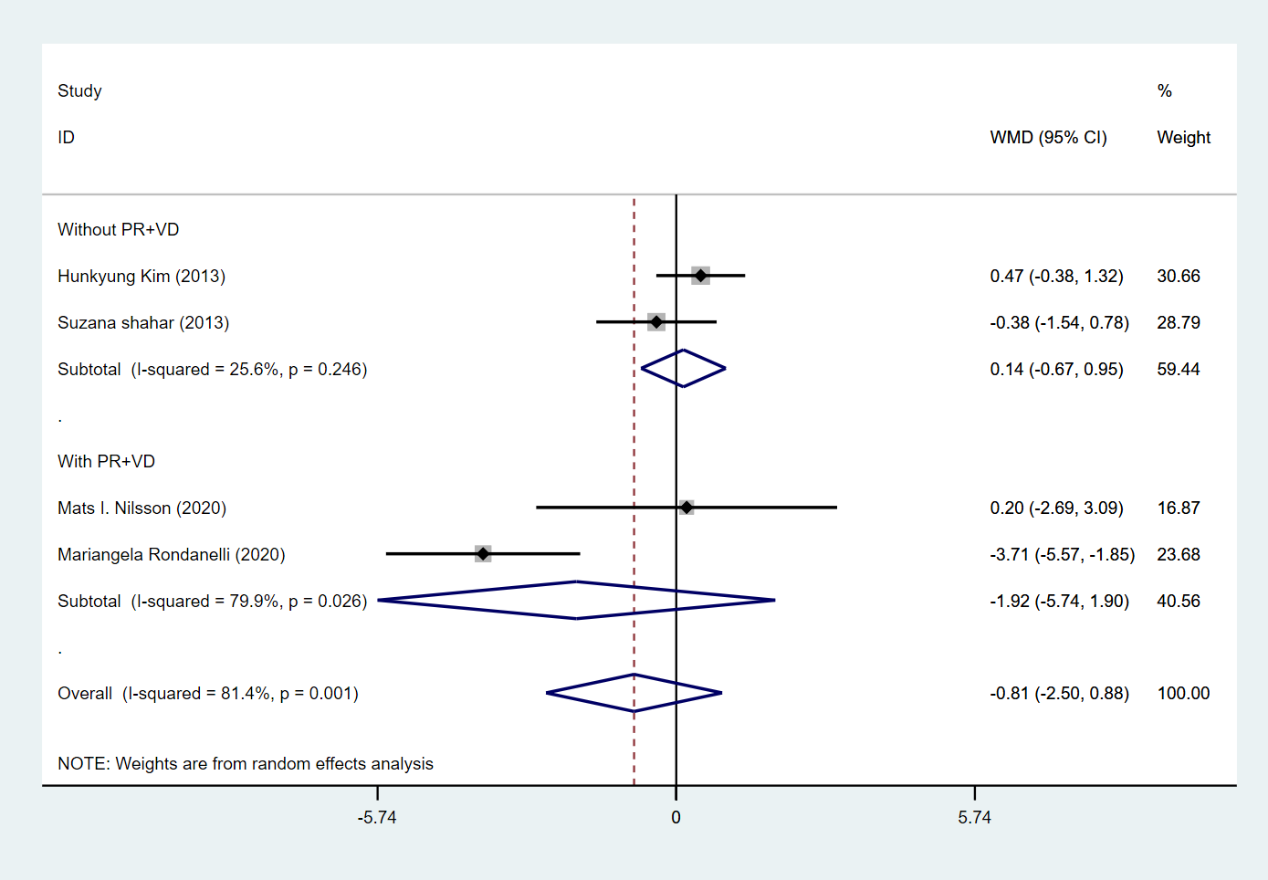


**Supplementary Figure 13** **|** TUG forest plot of resistance training combined with nutritional supplementation vs. resistance training alone in the with or without PR+VD subgroup. Overall estimates were obtained from forest plots of the meta-analysis using the random-effects model. Diamond icons and horizontal bars represent the overall estimate and 95% CI. WMD, weighted mean difference; CI, confidence interval.


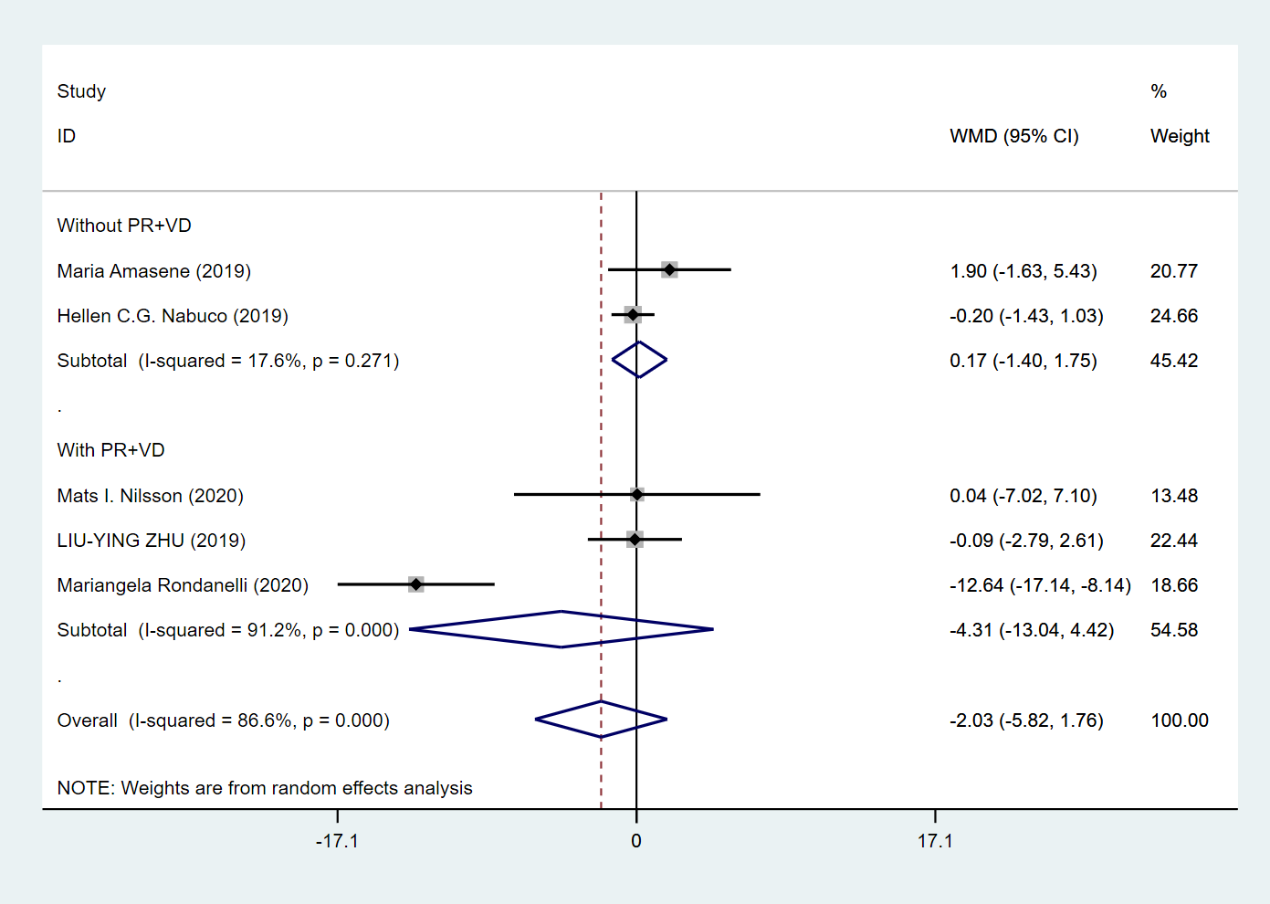


**Supplementary Figure 14** **|** Sit-stand test forest plot of resistance training combined with nutritional supplementation vs. resistance training alone in the with or without PR+VD subgroup. Overall estimates were obtained from forest plots of the meta-analysis using the random-effects model. Diamond icons and horizontal bars represent the overall estimate and 95% CI. WMD, weighted mean difference; CI, confidence interval.


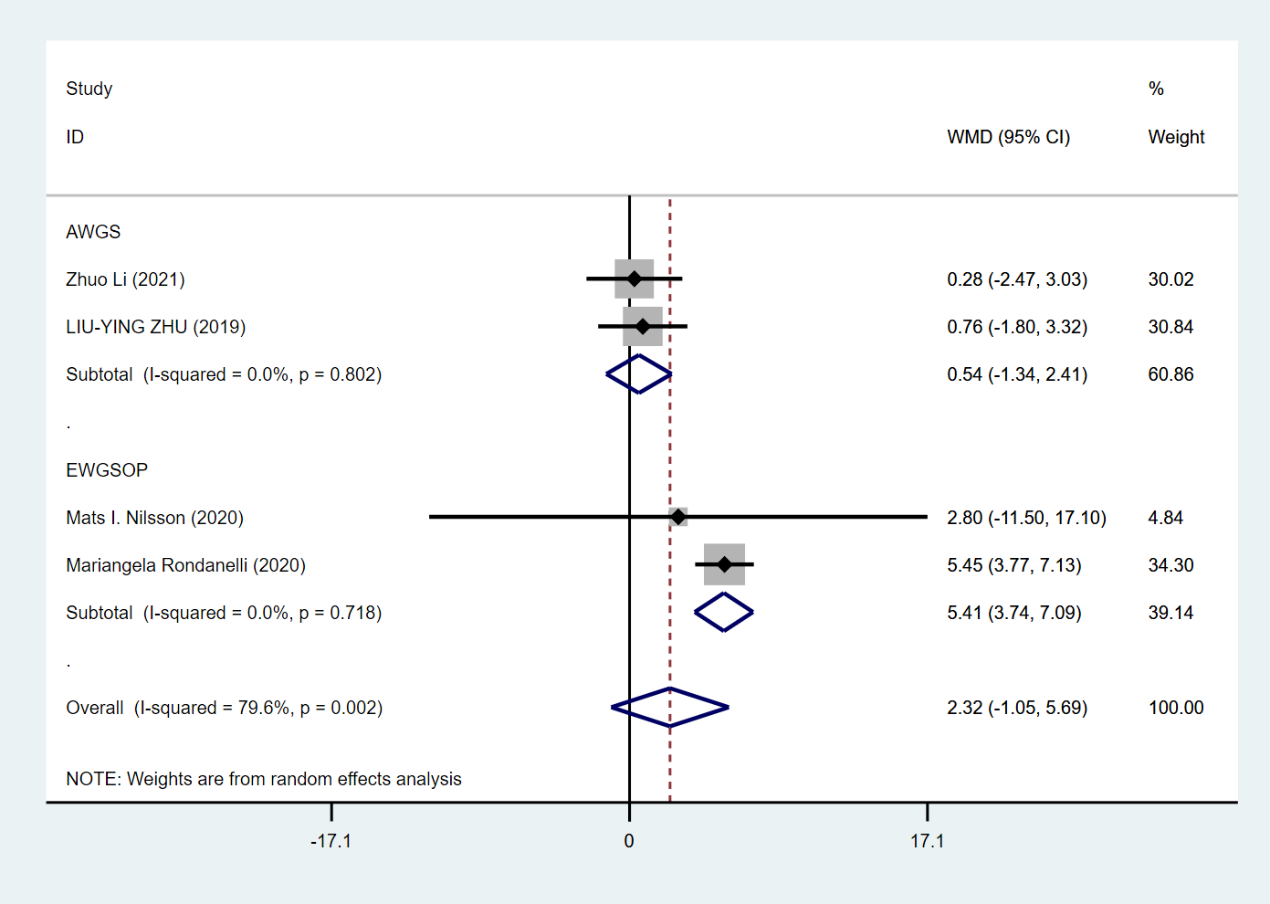


**Supplementary Figure 15** **|** Grip strength forest plot of resistance training combined with nutritional supplementation vs. resistance training alone in different diagnostic criteria. Overall estimates were obtained from forest plots of the meta-analysis using the random-effects model. Diamond icons and horizontal bars represent the overall estimate and 95% CI. WMD, weighted mean difference; CI, confidence interval.


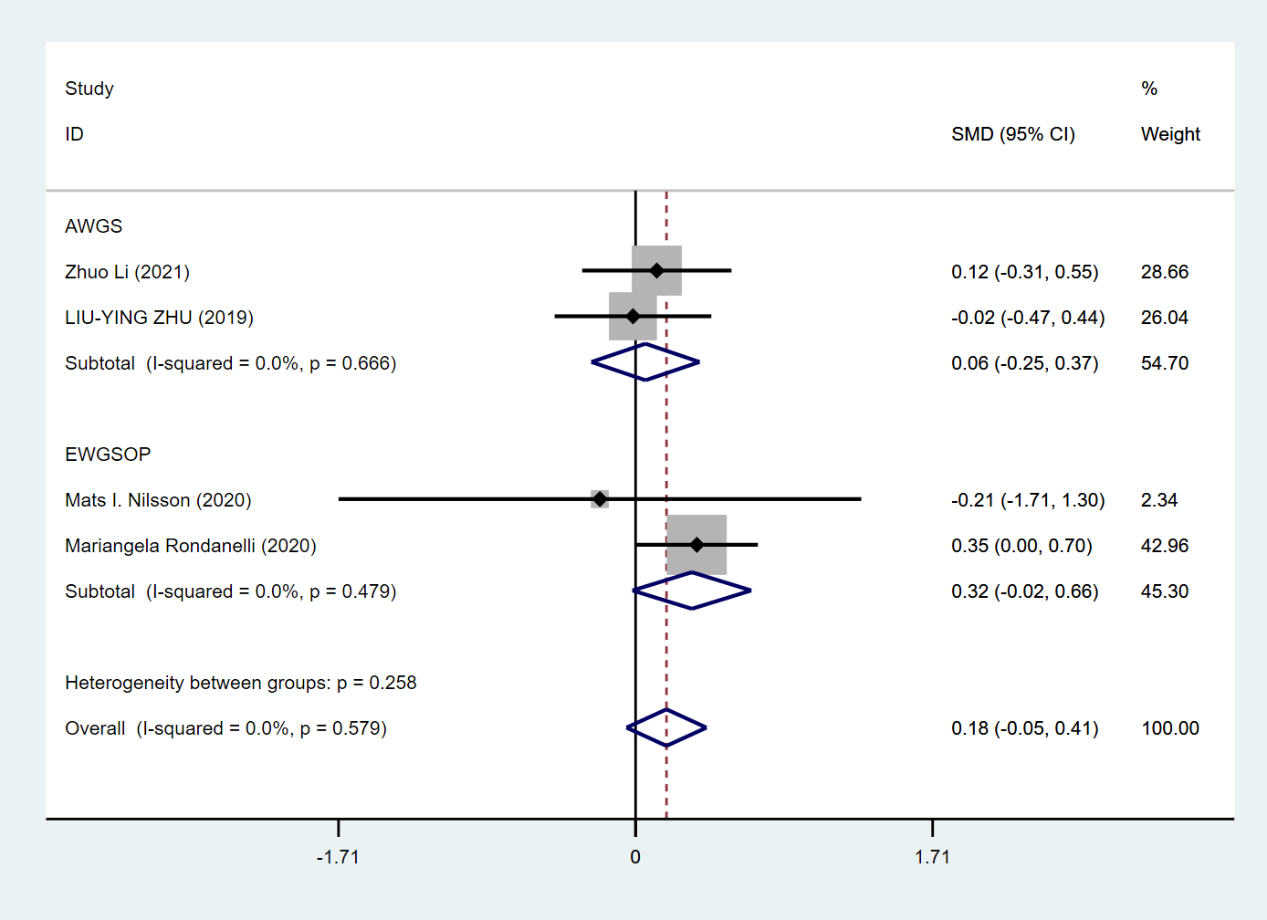


**Supplementary Figure 16** **|** Forest plot for skeletal muscle index of resistance training combined with nutritional supplementation vs. resistance training alone in different diagnostic criteria. Overall estimates were obtained from forest plots of the meta-analysis using the fixed-effects model. Diamond icons and horizontal bars represent the overall estimate and 95% CI. SMD, standardized mean difference; CI, confidence interval.


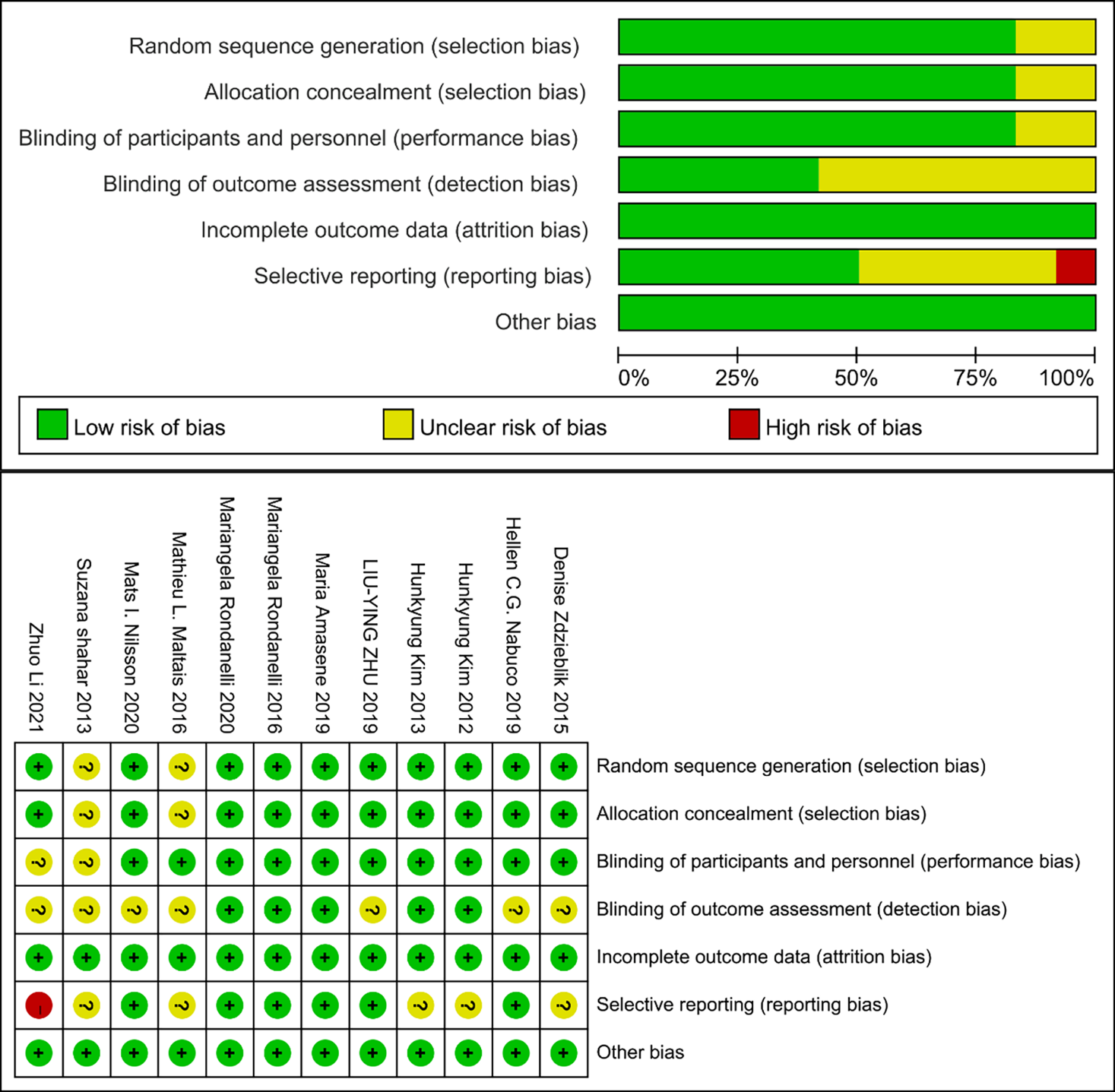


**Supplementary Figure 17** **|** Illustration of risk of bias assessment.
